# Selective DNA-PKcs inhibition extends the therapeutic index of localized radiotherapy and chemotherapy

**DOI:** 10.1172/JCI127483

**Published:** 2019-11-25

**Authors:** Catherine E. Willoughby, Yanyan Jiang, Huw D. Thomas, Elaine Willmore, Suzanne Kyle, Anita Wittner, Nicole Phillips, Yan Zhao, Susan J. Tudhope, Lisa Prendergast, Gesa Junge, Luiza Madia Lourenco, M. Raymond V. Finlay, Paul Turner, Joanne M. Munck, Roger J. Griffin, Tommy Rennison, James Pickles, Celine Cano, David R. Newell, Helen L. Reeves, Anderson J. Ryan, Stephen R. Wedge

**Affiliations:** 1 Cancer Research UK Newcastle Drug Discovery Unit, Translational and Clinical Research Institute, Newcastle University Centre for Cancer, Faculty of Medical Sciences, Newcastle University, Newcastle upon Tyne, United Kingdom.; 2 Cancer Research UK and UK Medical Research Council Oxford Institute for Radiation Oncology, Department of Oncology, University of Oxford, Oxford, United Kingdom.; 3 Medicinal Chemistry, Oncology, IMED Biotech Unit, AstraZeneca, Cambridge, United Kingdom.; 4 Astex Pharmaceuticals, Cambridge, United Kingdom.; 5 Cancer Research UK Newcastle Drug Discovery Unit, Chemistry, School of Natural and Environmental Sciences, Newcastle University, Newcastle upon Tyne, United Kingdom.; 6 Hepatopancreatobiliary Multidisciplinary Team, Freeman Hospital, Newcastle upon Tyne Hospitals NHS Foundation Trust, Newcastle upon Tyne, United Kingdom.

**Keywords:** Oncology, Therapeutics, DNA repair, Liver cancer, Radiation therapy

## Abstract

Potentiating radiotherapy and chemotherapy by inhibiting DNA damage repair is proposed as a therapeutic strategy to improve outcomes for patients with solid tumors. However, this approach risks enhancing normal tissue toxicity as much as tumor toxicity, thereby limiting its translational impact. Using NU5455, a newly identified highly selective oral inhibitor of DNA-dependent protein kinase catalytic subunit (DNA-PKcs) activity, we found that it was indeed possible to preferentially augment the effect of targeted radiotherapy on human orthotopic lung tumors without influencing acute DNA damage or a late radiation-induced toxicity (fibrosis) to normal mouse lung. Furthermore, while NU5455 administration increased both the efficacy and the toxicity of a parenterally administered topoisomerase inhibitor, it enhanced the activity of doxorubicin released locally in liver tumor xenografts without inducing any adverse effect. This strategy is particularly relevant to hepatocellular cancer, which is treated clinically with localized drug-eluting beads and for which DNA-PKcs activity is reported to confer resistance to treatment. We conclude that transient pharmacological inhibition of DNA-PKcs activity is effective and tolerable when combined with localized DNA-damaging therapies and thus has promising clinical potential.

## Graphical abstract

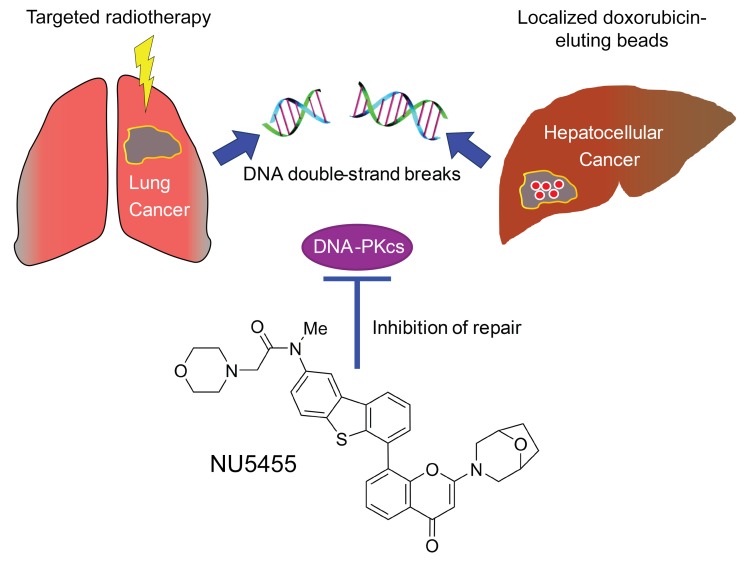

## Introduction

The activity of DNA-dependent protein kinase (DNA-PK) is essential to the repair of DNA double-strand breaks (DNA-DSBs) via canonical nonhomologous end joining (NHEJ) (1, 2). This repair mechanism involves recruitment of the DNA-PK holoenzyme to DNA-DSBs by two Ku70 and Ku80 heterodimeric subunits, which tethers the free DNA duplex strands together (3). A series of regulatory phosphorylation events, orchestrated by the serine/threonine protein kinase activity of the DNA-PK catalytic subunit (DNA-PKcs), restricts processing of the DNA (4), promotes activation of additional repair proteins required for DNA ligation (5, 6), and regulates the subsequent dissociation of DNA-PK from the Ku heterodimer (7). In contrast to the higher-fidelity repair of DNA-DSBs by homologous recombination, NHEJ occurs throughout the cell cycle and can function as a more rapid repair process to limit chromosomal translocations (8, 9).

Targeting of DNA-PK catalytic activity has been proposed as a strategy to augment the antitumor activity of clinically used therapies that result in DNA-DSBs, such as treatment with topoisomerase II inhibitors (e.g., doxorubicin or etoposide) or radiotherapy (10, 11). This rationale is also predicated on the fact that DNA-PK activation and gene expression have been shown to be elevated in a number of tumor types, including chronic lymphocytic leukemia, ovarian cancer, and hepatocellular cancer (HCC), and correlate with a poor outcome to standard therapies (12–14). A potential concern with this approach, however, is whether inhibition of DNA-PK catalytic activity will exacerbate the normal tissue toxicity of DNA-damaging agents to a level that necessitates a reduction in the standard-of-care treatment, thereby limiting the potential of the combination to afford an increased therapeutic outcome. This apprehension is founded in the knowledge that all nucleated cells contain the proteins required for NHEJ and that extreme sensitivity has been previously encountered (including fatalities) when leukemic patients with genetic deficiencies in the core NHEJ component DNA ligase IV have been treated with either myeloablative preconditioning therapy or prophylactic cranial irradiation (15, 16). Presently, it remains unclear whether transient pharmacological inhibition of DNA-PKcs can be harnessed to provide an increased therapeutic index.

We report the identification of NU5455, a novel highly selective orally bioavailable inhibitor of DNA-PKcs activity. NU5455 was used to evaluate pharmacological DNA-PKcs inhibition in combination with ionizing radiation (IR) or topoisomerase II inhibitors, by examining the responses of both tumor and normal tissues to treatment. Encouragingly, the data indicate that selective and transient DNA-PKcs inhibition in vivo can favorably augment an IR antitumor response without significantly affecting the resolution of acute DNA damage in normal tissues or exacerbating a late radiation-induced toxicity. In addition, we highlight the potential of using an oral DNA-PKcs inhibitor to enhance the antitumor activity of localized doxorubicin-eluting beads without an adverse effect on the host: an approach that has translational potential for the treatment of HCC.

## Results

### NU5455 selectively inhibits DNA-PK activity and DNA-DSB repair.

**Figure 1:**
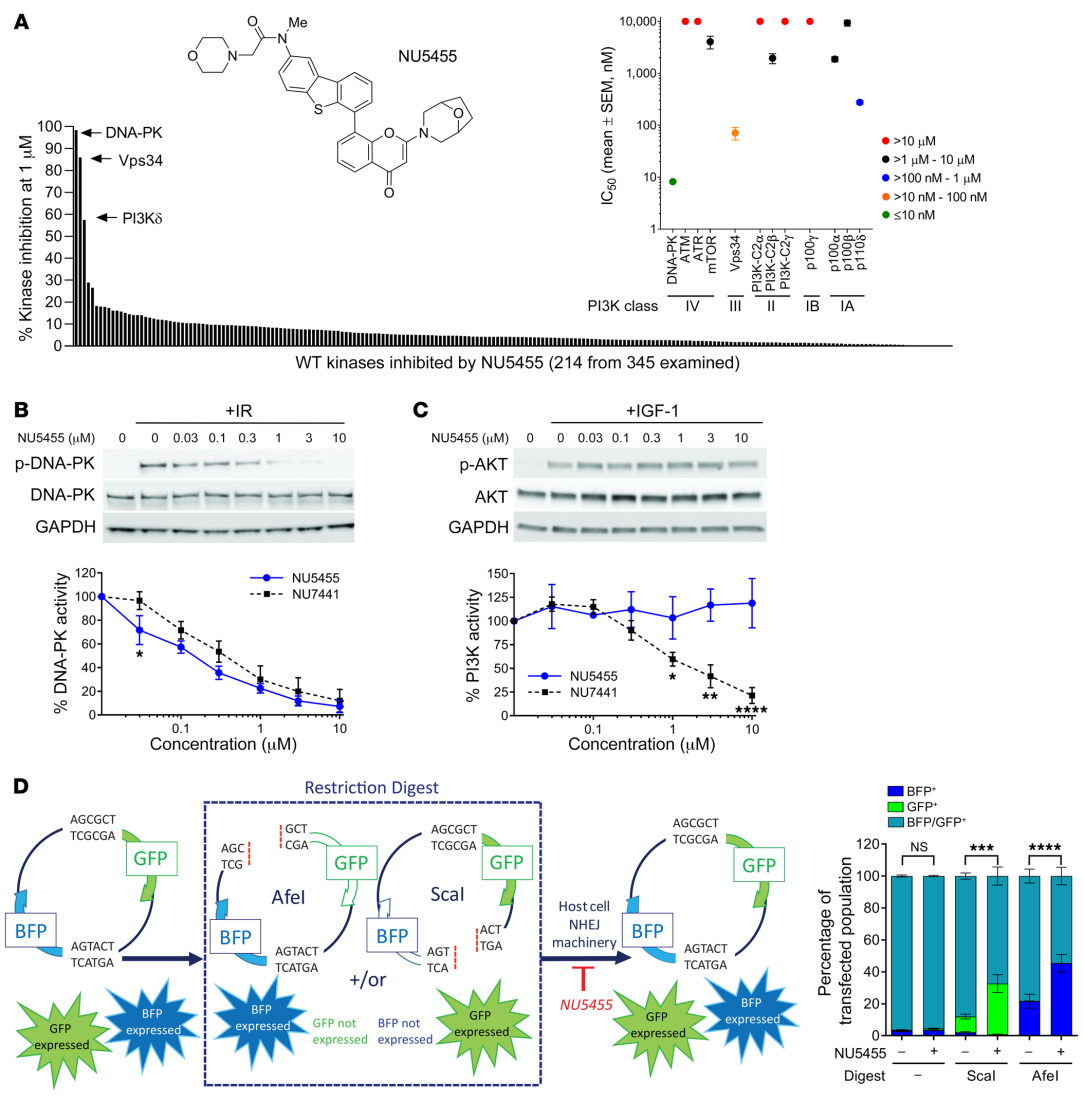
NU5455 is a selective inhibitor of DNA-PKcs activity. (**A**) Chemical structure of the DNA-PK inhibitor NU5455 (molecular weight = 595.71). The in vitro potency of NU5455 against DNA-PK and other kinases was assessed by SelectScreen Profiling (Life Technologies). Data depicted graphically represent the cell-free NU5455 IC_50_ values for PI3K family members (mean ± SEM, *n* = 4–7) and inhibitory activity of 1 μM NU5455 when tested against a panel of 345 wild-type kinases. (**B** and **C**) Changes in phospho–DNA-PK Ser2056 and phospho–AKT Ser473 30 minutes after treatment with 10 Gy IR or 50 ng/mL IGF-1, respectively, in MCF7 cells pretreated with vehicle, NU5455, or NU7441 for 1 hour. Percentage activity was determined relative to total DNA-PK or AKT using densitometry. (**D**) Plasmid repair assay enabling quantification of NHEJ-mediated DSB repair in HEK293T cells by measurement of the relative proportions of BFP and GFP. Cells were transfected with intact or linearized (AfeI or ScaI restriction endonuclease–treated) plasmid DNA and treated with NU5455 for 24 hours. With the exception of the broad kinase panel screen, all data represent the mean ± SEM from 4–7 (**A**) and 3 (**B**–**D**) independent experiments. Statistical significance was assessed using unpaired *t* tests (**B** and **C**) and 2-way ANOVA (**D**). *P < 0.05, **P < 0.01, ***P < 0.001, ****P < 0.0001.

NU5455 (Figure 1A and Supplemental Figure 1; supplemental material available online with this article; https://doi.org/10.1172/JCI127483DS1) was tested at 1 μM against 345 wild-type and 56 mutant kinases (Life Technologies SelectScreen), and found to be a highly selective ATP-competitive kinase inhibitor: the kinase activity of DNA-PKcs, Vps34, and PI3Kδ was inhibited by 98%, 86%, and 57%, respectively, but little inhibitory activity was demonstrated against the remaining 398 kinases, with less than 10% inhibition in the majority and 20%–29% inhibition evident in only 2 others (Supplemental Table 1). IC_50_ determinations against each of the PI3K family members further verified that NU5455 was a potent inhibitor of DNA-PKcs activity (IC_50_ of 8.2 ± 2 nM; Figure 1A and Supplemental Table 2), with selectivity versus Vps34 (8.7-fold), PI3Kδ (33.7-fold), and ATM and ATR (both >1200-fold). Importantly, NU5455 demonstrated a 228-fold selectivity margin for DNA-PKcs kinase activity versus that of PI3Kα, activity against the latter being evident with a number of earlier DNA-PKcs inhibitors (e.g., KU60648; ref. 17), which is preferably avoided, given that PI3Kα signaling has a key cardioprotective role (18). This activity and selectivity were also evident at a cellular level. NU5455 inhibited radiation-induced activation of DNA-PK with an IC_50_ of 168 nM (Figure 1B) but did not inhibit IGF-stimulated activation of AKT in MCF7 cells even at 10 μM (Figure 1C), which is mediated by IGF-1R activation of the p85 regulatory subunit of PI3K (19). In contrast, while our widely used prototype DNA-PKcs inhibitor NU7441 demonstrated similar activity against DNA-PK activation in MCF7 cells (IC_50_ of 405 nM), it also inhibited an IGF/PI3K-stimulated signaling response by 40% ± 7% at 1 μM (Figure 1, B and C, and Supplemental Figure 2).

To examine the mechanistic consequences of NU5455 treatment for DNA-DSB repair, HEK293T cells were transfected with a dual BFP- and GFP-containing reporter construct that enabled quantification of the repair of DNA-DSBs generated following treatment with either AfeI or ScaI restriction endonucleases. NU5455 (1 μM) was found to inhibit the repair of DNA-DSBs induced by treatment with either enzyme within a 24-hour period (Figure 1D and Supplemental Figure 3). In addition, phosphorylation of histone H2AX (γH2AX) and the formation of 53BP1 foci were quantified in Calu-6 and A549 human lung cancer cells as early biomarkers of DNA-DSB formation, following 10 Gy of radiation treatment in the presence and absence of NU5455 (5 μM). Treatment with NU5455 led to a significant increase in the number of colocalized γH2AX and 53BP1 foci observed at 5 hours after irradiation (Supplemental Figure 4).

Collectively these data indicate NU5455 to be a highly selective inhibitor of DNA-PKcs that is active in cells and that can perturb DNA-DSB repair by NHEJ.

### NU5455 is an effective radiosensitizer in vitro.

**Figure 2:**
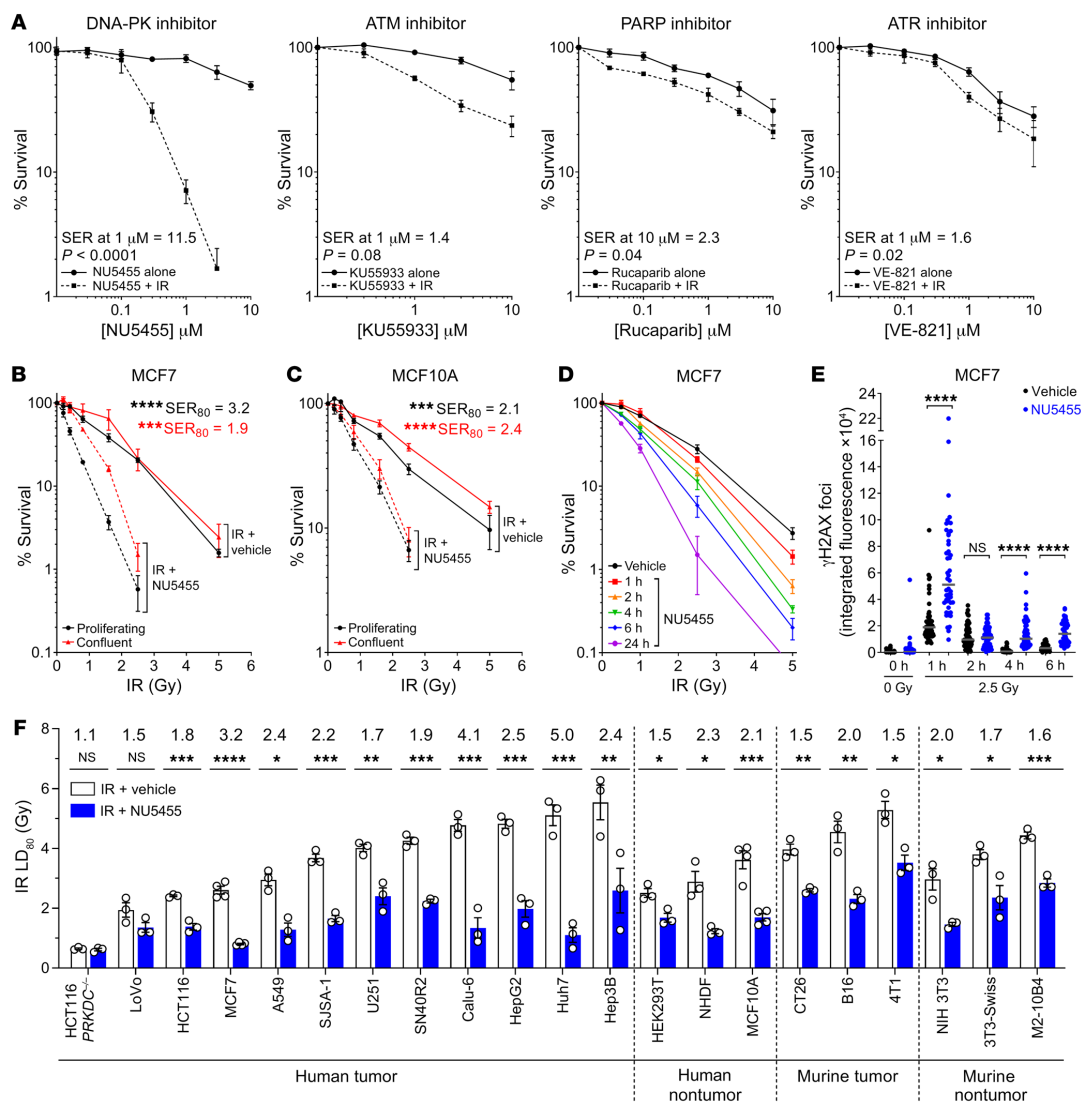
NU5455 is an effective radiosensitizer in vitro. (**A**) Clonogenic survival of MCF7 cells pretreated with NU5455, the ATM inhibitor KU55933, the PARP inhibitor rucaparib, or the ATR inhibitor VE-821 for 1 hour before IR (2 Gy). Clonogenic assays involved continued incubation with compounds prior to reseeding of cells into drug-free media 24 hours after irradiation. SER, sensitization enhancement ratio. (**B** and **C**) MCF7 (**B**) and MCF10A (**C**) clonogenic survival under confluent or exponentially growing conditions. Cells were treated with vehicle or NU5455 (1 μM) for 1 hour before irradiation and reseeding 24 hours after irradiation. SER_80_ indicates the SER between radiation doses with and without NU5455 that induced an 80% inhibition of clonogenic cell survival (LD_80_). (**D**) Clonogenic survival of MCF7 cells pretreated with NU5455 (1 μM) for 1 hour before irradiation, and for a further 1, 2, 4, 6, or 24 hours after irradiation before incubation in drug-free media prior to reseeding at 24 hours. (**E**) Integrated total nuclear fluorescence of γH2AX foci in MCF7 cells pretreated with NU5455 (1 μM) for 1 hour and fixed 0–24 hours after irradiation (2.5 Gy). At least 50 cells were analyzed per treatment group. (**F**) LD_80_ values from clonogenic survival assays of cell lines treated with vehicle or NU5455 (1 μM) for 1 hour before, and 24 hours after, irradiation. Fold potentiation is shown above each cell line. All graphs (**A**–**F**) represent the mean ± SEM from at least 3 independent experiments, with the exception of graph **E**, which is a single representative of 2 independent experiments (see also Supplemental Figure 6). Statistical significance was assessed using unpaired *t* tests, with the exception of graph **E**, for which Mann-Whitney *U* tests were used. *P < 0.05, **P < 0.01, ***P < 0.001, ****P < 0.0001.

We examined the ability of NU5455 to enhance a 2-Gy dose of IR in comparison with treatment with inhibitors of other DNA repair enzymes — namely KU55933, which inhibits ATM serine/threonine kinase (a DNA-DSB repair checkpoint that activates a range of proteins including p53 and Chk2) (20); rucaparib, which inhibits poly(ADP-ribose) polymerase (PARP; involved in DNA single-strand repair) (21); and VE-821, which inhibits ATR serine/threonine kinase (involved in DNA single-strand break repair and activation of Chk1) (22). Each inhibitor was studied in MCF7 breast tumor cells over a range that included concentrations previously shown to be pharmacologically active (10 μM KU55933 [ATM], 0.4 μM rucaparib [PARP], and 1 μM VE-821 [ATR]) (20–22). While the clonogenic cell killing induced by treatment with 2 Gy IR was further enhanced by treatment with the relevant concentrations of an ATM or ATR inhibitor (KU55933, 2.3-fold at 10 μM [*P* = 0.04]; VE-821, 1.6-fold at 1 μM [*P* = 0.02]), the potential radio-enhancement observed with the PARP inhibitor did not quite reach statistical significance (1.4-fold at 1 μM [*P* = 0.08]). In comparison, combination therapy with NU5455 had a significantly more profound effect, with NU5455 monotherapy potentiating the effect of 2 Gy IR 11.5-fold at 1 μM and 38-fold at 3 μM (both *P* = 0.0001 respectively; Figure 2A).

The formation of DNA-DSBs can be induced either through direct radiation damage or from stalled DNA replication forks that can persist into S phase (23) and that may potentially be more evident in tumor cells with a compromised G_1_/S checkpoint. While the repair of DNA-DSBs by NHEJ is suggested to occur throughout the cell cycle (24), this may also be influenced by replication, representing the major repair pathway for radiation-induced DNA-DSBs during G_1_ (25). To examine the influence of cellular proliferation on DNA-DSB repair following irradiation and NU5455 treatment, the clonogenic survival of MCF7 tumor cells was measured either in cells undergoing normal exponential growth or in those that had been allowed to reach confluence before treatment and in which an increased G_0_/G_1_ fraction (from 29.1% ± 4.9% to 77.8% ± 2.9%) and diminished S phase fraction (from 42.0% ± 3.6% to 6.6% ± 2.3%) were observed (*n* = 4, mean ± SEM; Supplemental Figure 5A). The irradiation of confluent MCF7 cells did not appear to markedly influence the repair of potentially lethal damage (26), as their intrinsic radiosensitivity was not significantly different from that of proliferating cells at any dose of IR examined (Figure 2B; *P* = 0.13–0.92). In contrast, proliferating MCF7 cells were more sensitive than confluent cells to treatment with the combination of NU5455 and IR, at 0.4 Gy (*P* = 0.009), 0.8 Gy (*P* = 0.00002), and 1.6 Gy (*P* = 0.0003) (Figure 2B). Experiments were also conducted in nontumorigenic MCF10A normal breast epithelial cells (27), using confluence conditions that increased the G_0_/G_1_ fraction from 62% ± 7.9% to 95% ± 1.6% (*n* = 4, mean ± SEM; Supplemental Figure 5B). Confluent MCF10A cells were found to be more intrinsically radioresistant than proliferating cells at 1.6 and 2.5 Gy (*P* = 0.03 and 0.01, respectively; Figure 2C). However, while NU5455 treatment enhanced the activity of IR in MCF10A cells, the clonogenic survival of confluent and proliferating MCF10A cells following combination treatment did not differ significantly (Figure 2C; *P* > 0.2 at all IR doses). Collectively, these data suggest that the combination of IR and NU5455 can induce more pronounced cell killing in proliferating, as opposed to confluent, tumor cells, but that the response to combination treatment, and influence of cell cycle phase, may be cell type–dependent.

We also explored the effect of NU5455 (1 μM) treatment duration on the sensitivity of MCF7 cells to IR. The ability of NU5455 to enhance radiation-induced clonogenic survival was highly dependent on incubation time; LD_80_ values indicated 1 hour of NU5455 treatment to produce only a marginal effect that did not reach statistical significance (1.2-fold, *P* = 0.09), 4- to 6-hour treatment to enhance the radiation response significantly (1.5- to 1.7-fold, *P* < 0.002), and 24-hour treatment to elicit further radio-enhancement (2.4-fold, *P* <0.0001) (Figure 2D). An analysis of γH2AX foci following 2.5 Gy irradiation in MCF7 cells revealed that incubation with NU5455 enhanced the number of foci formed within 1 hour of irradiation (Figure 2E and Supplemental Figure 6). While some resolution of the γH2AX foci was evident in cells either without or with continuous NU5455 treatment, the number of foci in the NU5455-treated cells remained elevated even at 24 hours (Figure 2E and Supplemental Figure 6).

The radiosensitizing effect of NU5455 treatment was further examined in different cell lines. This included HCT116 colorectal carcinoma DNAPK^–/–^ cells, in which the effect of radiation was not augmented by treatment with NU5455 (1 μM), in contrast to isogenic HCT116 parental cells, in which a significant radiosensitizing effect was observed (Figure 2F and Supplemental Figure 7). Similarly, concomitant NU5455 (5 μM) treatment did not alter the response of human chronic myeloid leukemia HAP-1 DNA-PK–null cells to IR, but markedly increased the clonogenic cell-killing effect of 1–4 Gy IR in HAP-1 cells that express 1 copy of *PRKDC* (Supplemental Figure 8). These data are consistent with the radiosensitization effect of NU5455 being attributable to the inhibition of DNA-PKcs activity.

NU5455 treatment (1 μM, 24 hours) was also examined in cells representative of lung (A549, Calu-6) and colorectal cancer (HCT116, LoVo), osteosarcoma (SJSA-1), glioblastoma (U251), and HCC (Hep3B, HepG2, Huh7). While the tumor cell lines selected exhibited different intrinsic radiosensitivities and had differences in *TP53* status (wild-type, mutant nonfunctional, and deleted; Supplemental Table 3), NU5455 enhanced the effect of IR significantly in all DNA-PK–expressing cells with the exception of LoVo, SER_80_ values (the sensitizing enhancement ratio 80, which is the ratio between radiation doses with and without NU5455 that induced an 80% inhibition of clonogenic cell survival) ranging from 1.5- to 5.0-fold (median 2.3-fold; Figure 2F and Supplemental Figure 7). A comparable level of radio-potentiation was also observed in 3 human noncancer cell lines (1.5- to 2.3-fold SER_80_) (Figure 2F and Supplemental Figure 9). Similarly, NU5455 was found to enhance the effect of IR in both mouse tumor and normal fibroblast cell lines (1.5- to 2.0-fold SER_80_) (Figure 2F and Supplemental Figure 10). These data verify that NU5455 can significantly increase the radiosensitivity of DNA-PK–expressing tumor and normal/fibroblast cells of both human and murine origin in vitro.

### NU5455 augments external beam radiotherapy in subcutaneous lung tumor xenografts.

**Figure 3:**
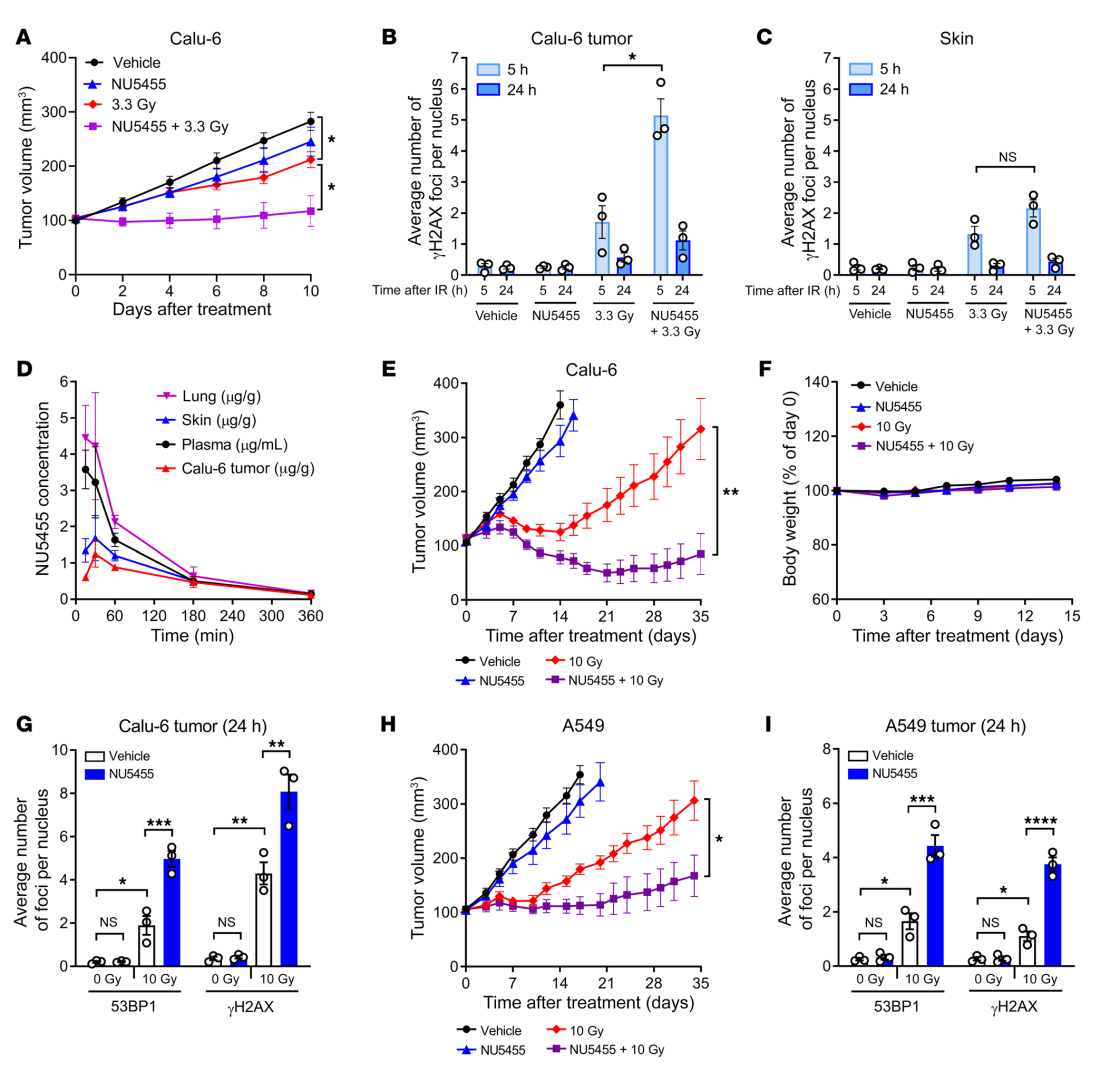
NU5455 preferentially augments radiotherapy in subcutaneous Calu-6 and A549 lung tumor xenografts versus surrounding skin. (**A**) Mean tumor volume of mice bearing Calu-6 subcutaneous xenografts treated with NU5455 (30 mg/kg orally) or vehicle 30 minutes before localized radiation (0 Gy or 3.3 Gy, 4–5 mice per group). (**B** and **C**) Number of γH2AX foci per nucleus in subcutaneous Calu-6 tumors (**B**) and the surrounding skin (**C**) collected 5 and 24 hours after irradiation (3 mice per group). (**D**) NU5455 concentrations in the plasma, lung, skin, and subcutaneous Calu-6 tumors 15–360 minutes after oral administration of NU5455 (30 mg/kg, 3 mice per group). (**E**) Volume of subcutaneous Calu-6 tumors following treatment with NU5455 (30 mg/kg orally) or vehicle 30 minutes after localized radiation (0 Gy or 10 Gy, 4–6 mice per group). (**F**) Corresponding percentage change in body weight of mice bearing Calu-6 tumors. (**G**) Number of 53BP1 and γH2AX foci per nucleus in Calu-6 tumors collected 24 hours after irradiation (3 mice per group). (**H**) Mean tumor volume from mice bearing A549 subcutaneous xenografts treated with NU5455 (30 mg/kg orally) or vehicle 30 minutes before localized radiation (0 Gy or 10 Gy, 4–5 mice per group). (**I**) Number of 53BP1 and γH2AX foci per nucleus in A549 tumors collected 24 hours after irradiation (3 mice per group). All graphs represent the mean ± SEM. Statistical significance was assessed using unpaired *t* tests, with the exception of **G** and **I**, for which it was assessed by 1-way ANOVA. *P < 0.05, **P < 0.01, ***P < 0.001, ****P < 0.0001.

To determine whether the radio-enhancing activity of NU5455 could also be observed in vivo, Calu-6 and A549 human non–small cell lung cancer tumor xenografts were established subcutaneously (approximately 100 mm^3^ volume) in athymic mice and treated with a single oral dose of either NU5455 (30 mg/kg) or the corresponding vehicle, 30 minutes before a single dose of tumor-localized irradiation. While a single dose of 3.3 Gy radiation produced a relatively marginal inhibition of Calu-6 tumor growth, the concurrent administration of NU5455 enhanced the antitumor activity significantly to induce a cytostatic response for 6 days following treatment (mean tumor volume = 104 mm^3^ at day 0 vs. 102 mm^3^ at day 6) (Figure 3A). As antibodies against human phospho–DNA-PK (Ser2056) do not cross-react with the complementary phosphorylation site in murine DNA-PK (Ser2053) (28), further ex vivo comparisons between mouse skin and human tumor tissues were based on quantification of γH2AX, reflecting DNA-DSBs. A histological analysis of Calu-6 subcutaneous tumors indicated that the number of radiation-induced γH2AX foci remaining 5 hours after irradiation was 3.0-fold greater (*P* = 0.01) with concomitant NU5455 treatment (Figure 3B and Supplemental Figure 11A). In contrast, 5 hours after irradiation, the number of radiation-induced γH2AX foci in the skin immediately surrounding the tumor was augmented only 1.6-fold with NU5455 treatment (Figure 3C and Supplemental Figure 11A), a change that did not reach statistical significance (*P* = 0.09). These observations are likely to reflect a difference in the rate of repair of DNA-DSBs in the presence of NU5455, with radiation-induced DNA-DSBs being potentially less efficiently resolved in Calu-6 tumors versus skin. In addition, since Calu-6 tumor cells will have a greater proliferation rate than normal mouse skin, their replication past unrepaired DNA-DSBs could conceivably also contribute to additional γH2AX foci that are retained in the presence of NU5455. A pharmacokinetic analysis of NU5455 following administration of a 30-mg/kg oral dose of NU5455 to Calu-6 tumor–bearing mice indicated that the NU5455 concentrations in plasma, lung, tumor, or the surrounding skin were maintained at approximately 1 μM (i.e., 0.6 μg/mL or μg/g tissue) or above for a period of 3 hours (Figure 3D). Notably, the concentration of NU5455 in the tumor compartment was similar to that in the adjacent skin and did not exceed the latter at any time (Figure 3D).

When NU5455 was combined with a 10-Gy dose of radiation, regression of Calu-6 tumors was induced (Figure 3E), with 3 of 6 mice having no evidence of tumor at the end of the experiment (day 38). This enhanced antitumor response was achieved without any adverse effect or impact on body weight (Figure 3F). With this increased radiation dose, a greater number of γH2AX foci were evident in Calu-6 tumors 24 hours after treatment, which was elevated further by concurrent NU5455 administration (Figure 3G and Supplemental Figure 11B). The magnitude of 53BP1 staining was also increased when NU5455 was combined with 10 Gy of radiation, indicative of a greater engagement of repair by NHEJ (Figure 3G and Supplemental Figure 11B). NU5455 also enhanced the antitumor activity of a 10-Gy radiation treatment in the A549 lung tumor model (Figure 3H), which was similarly accompanied by an increase in the number of γH2AX foci and 53BP1 staining (Figure 3I and Supplemental Figure 11C). That the magnitude of radio-enhancement by NU5455 was less than in the Calu-6 model may reflect a cell-intrinsic difference between the 2 tumors that was also evident in vitro (Figure 2F).

### NU5455 augments external beam radiotherapy in orthotopic lung tumor xenografts but does not significantly enhance radiation-induced damage to normal lung.

**Figure 4:**
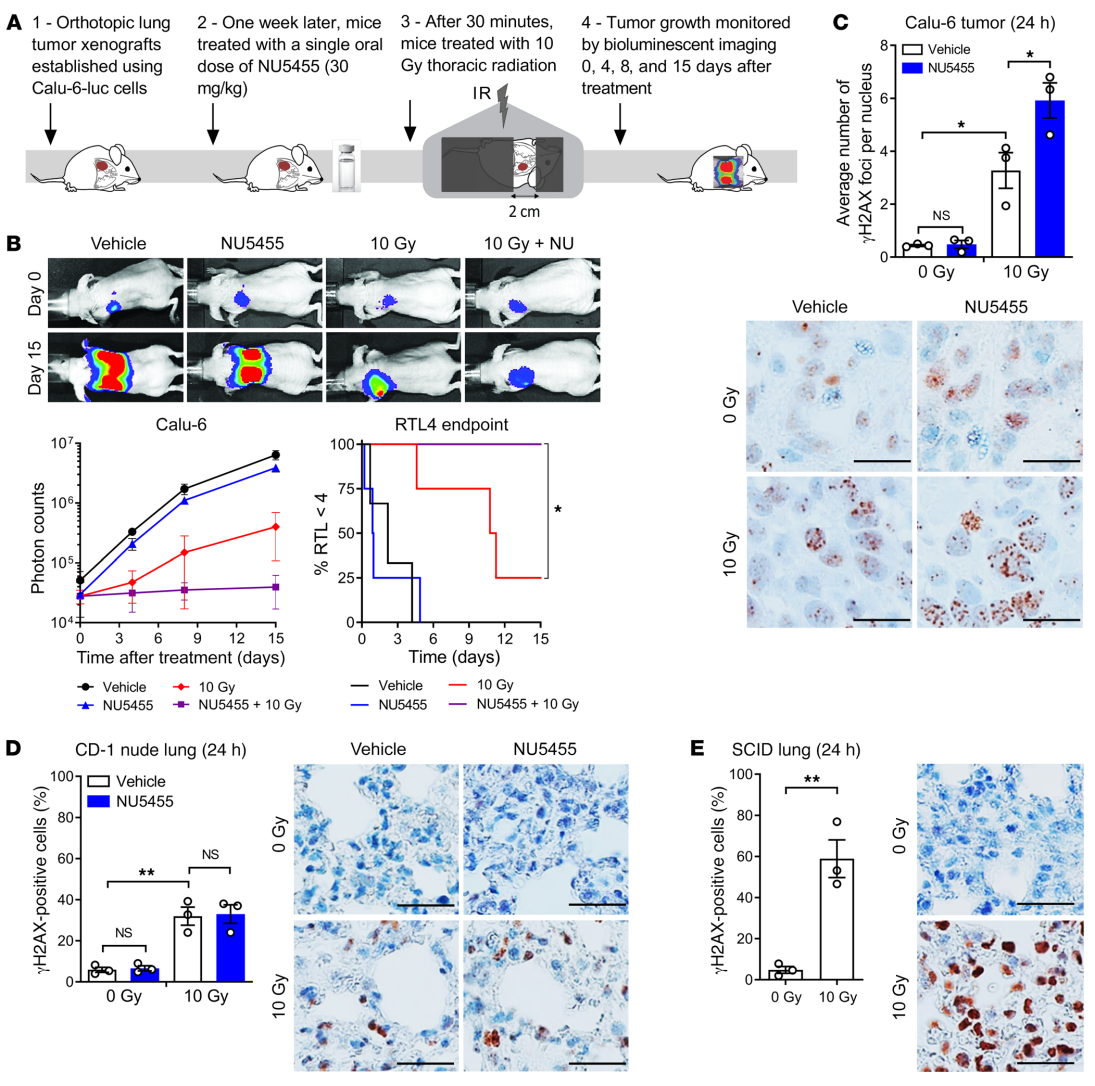
NU5455 augments the effect of radiotherapy in orthotopically implanted Calu-6 lung tumors but does not enhance acute radiation damage to the lung. (**A**) Luciferase-expressing Calu-6 cells were injected into the left lungs of immunocompromised mice. One week after injection, mice were treated with NU5455 (30 mg/kg orally) or vehicle 30 minutes before thoracic radiation (0 Gy or 10 Gy, 3–4 mice per group). Tumor growth was monitored by bioluminescent imaging 0, 4, 8, and 15 days after treatment. (**B**) Representative bioluminescent images at days 0 and 15, with mean photon counts from the thoracic region of mice over time, and the time taken to reach 4 times the tumor luminescence at the start of treatment (RTL4). (**C**) Number of γH2AX foci per nucleus in orthotopic Calu-6 tumors collected 24 hours after irradiation (3 mice per group), with representative immunohistochemistry images. (**D**) Percentage of γH2AX-positive cells in normal lung tissue 24 hours after irradiation in mice treated with NU5455 (30 mg/kg orally) or vehicle 30 minutes before mock irradiation or 10 Gy treatment (3 mice per group), with representative images. (**E**) Percentage of γH2AX-positive cells in normal lung tissue from SCID mice 24 hours after receiving mock irradiation or 10 Gy treatment (3 mice per group). Scale bars: 25 μm. All graphs represent the mean ± SEM. Statistical significance was assessed using a log-rank test (**B**), 1-way ANOVA (**C** and **D**), and an unpaired *t* test (**E**). *P < 0.05, **P < 0.01.

Luciferase-expressing Calu-6 cells were implanted orthotopically into the lungs of CD-1 nude mice and the effect of treatment with 10 Gy thoracic radiation with or without a single dose of NU5455 (30 mg/kg) ascertained by bioluminescent imaging (Figure 4A). NU5455 treatment enhanced the antitumor effect of the radiation, with a significant difference in the time taken to achieve a 4-fold increase in bioluminescent signal (Figure 4B and Supplemental Figure 12A), and was well tolerated (Supplemental Figure 12B). The number of γH2AX foci evident 24 hours after treatment was also increased significantly by NU5455 in the orthotopically implanted lung tumors (Figure 4C). Since the nuclei of mouse lung epithelial cells are too small to accurately quantify γH2AX foci, the percentage of γH2AX-positive normal lung cells following 10 Gy of thoracic irradiation was determined instead. An examination of the corresponding lungs that did not receive tumor implantation showed the number of γH2AX-positive cells to have been unaffected by NU5455 administration 24 hours after irradiation (Figure 4D). In contrast, in SCID mice, which express a truncated, catalytically inactive form of DNA-PK as a result of a nonsense mutation in *PRKDC* (29), the percentage of γH2AX-positive normal lung epithelial cells following 10 Gy of thoracic radiation was approximately double that observed in CD-1 nude mice (Figure 4E), indicating that constitutive deletion of the target protein does have a significant effect on the response of a normal tissue to radiation.

**Figure 5:**
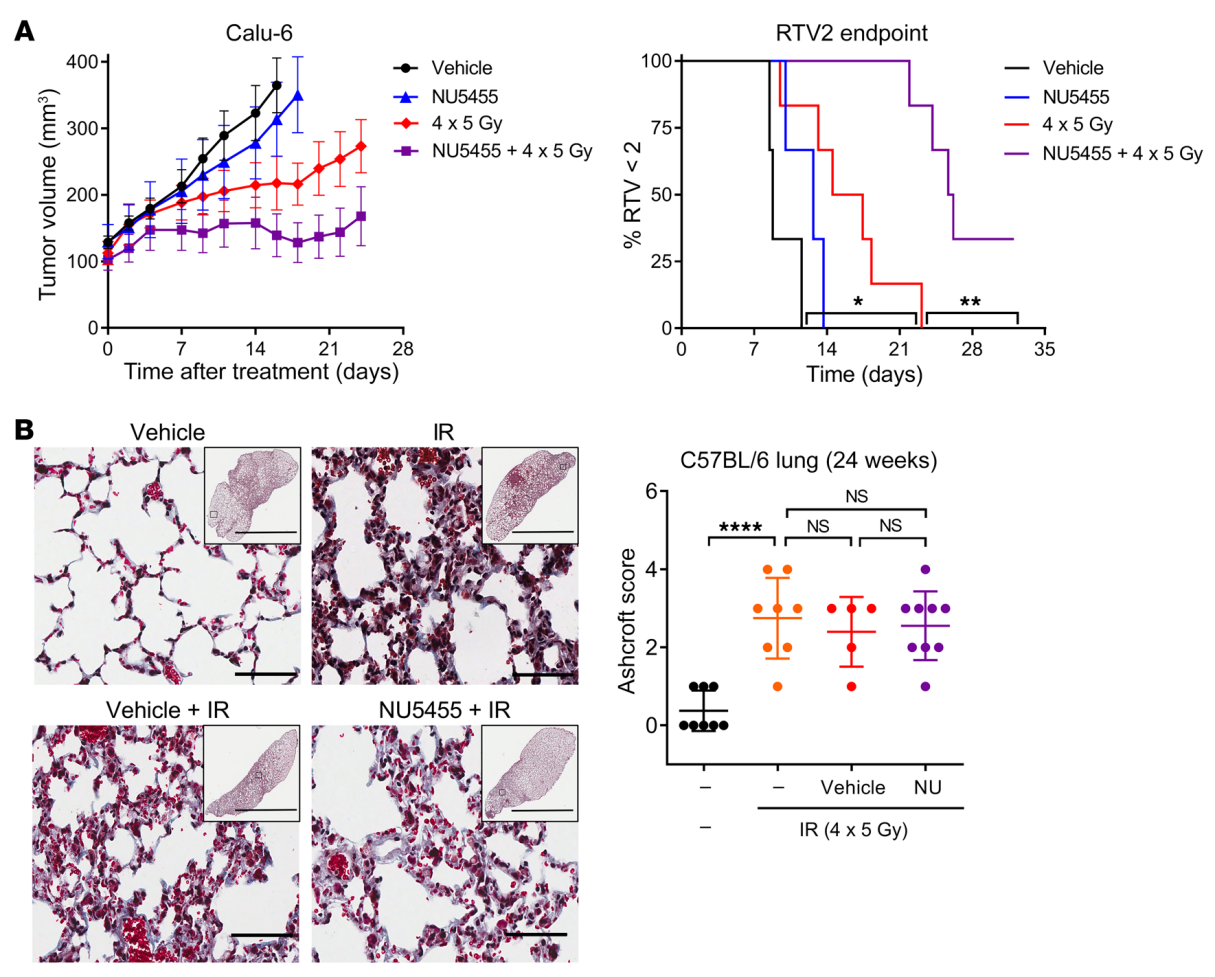
NU5455 augments the effect of fractionated radiotherapy in Calu-6 tumors without enhancing late radiation damage to the lung. (**A**) Mean tumor volume of mice bearing Calu-6 subcutaneous xenografts treated with localized radiation (4 × 5 Gy on days 1, 4, 7, and 10) with either NU5455 (30 mg/kg orally) or vehicle 30 minutes before and 5 hours after each dose of IR (3–5 mice per group). The time taken to reach 2 times the tumor volume at the start of treatment (RTV2) was also assessed. (**B**) Masson’s trichrome staining was used to evaluate lung fibrosis in mice 24 weeks after treatment with thoracic radiation (4 × 5 Gy on days 1, 4, 7, and 10) with or without NU5455 (30 mg/kg orally) or vehicle 30 minutes before and 5 hours after each dose of IR. Samples were scored using the modified Ashcroft system on a scale of 0 to 8 (5–9 mice per group). Representative images are depicted. Scale bars: 100 μm (main images) and 4 mm (inset images). All graphs represent the mean ± SEM. Statistical significance was assessed using a log-rank test (**A**) and 1-way ANOVA (**B**). *P < 0.05, **P < 0.01, ****P < 0.0001.

To examine the effect of NU5455 treatment on efficacy versus a late radiation toxicity, a total dose of 20 Gy of thoracic radiation was administered either to CD-1 nude mice bearing subcutaneous Calu-6 tumors or to non–tumor-bearing C57BL/6 mice, as four 5-Gy fractions given on days 1, 4, 7, and 10. This fractionated regimen was adapted from an existing method (30), resulting in focal areas of lung fibrosis at 24 weeks following treatment, but at a level that would enable any exacerbation of fibrosis to be readily detected. We also established that the plasma pharmacokinetic profile of NU5455 was comparable in CD-1 nude mice and C57BL/6 mice (Supplemental Figure 13A). Administration of 2 oral doses of 30 mg/kg NU5455 (30 minutes before and 5 hours after each radiation dose) was found to increase the time taken for human tumors in CD-1 nude mice to double in volume when compared with those treated with radiation and vehicle (Figure 5A). In contrast, the same NU5455 treatment regimen did not exacerbate radiation-induced lung fibrosis in C57BL/6 mice (Figure 5B).

### NU5455 sensitizes to topoisomerase II inhibitor treatment in vitro but has a narrow therapeutic index when combined with systemic chemotherapy in vivo.

**Figure 6:**
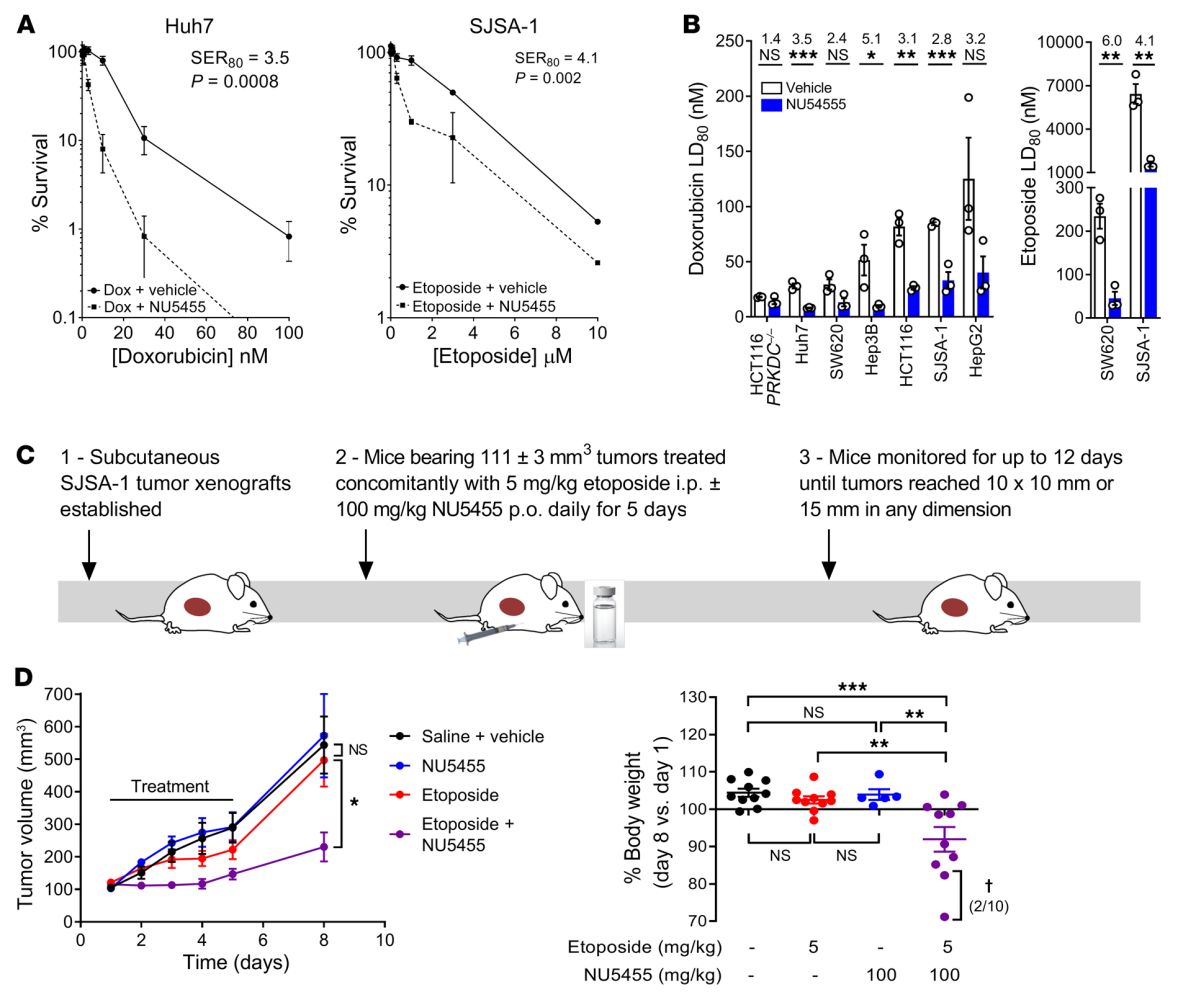
NU5455 sensitizes tumors to topoisomerase II inhibitors but has a narrow therapeutic index when combined with systemic chemotherapy in vivo. (**A**) Clonogenic survival of Huh7 and SJSA-1 cells pretreated with vehicle or NU5455 (1 μM) for 1 hour before the addition of doxorubicin or etoposide for 24 hours before replating into drug-free media. (**B**) LD_80_ values from clonogenic survival assays of human tumor cell lines pretreated with vehicle or NU5455 (1 μM) for 1 hour before the addition of doxorubicin or etoposide for 24 hours before reseeding into drug-free media. Fold potentiation is indicated for each cell line. **A** and **B** depict the mean ± SEM from 3 independent experiments. (**C**) Mice bearing subcutaneous SJSA-1 tumor xenografts (103–121 mm^3^) were treated with NU5455 (100 mg/kg orally) with or without etoposide (5 mg/kg, i.p.) daily for 5 days. (**D**) Mean SJSA-1 tumor volume, and percentage body weight at day 8 relative to pretreatment body weight on day 1 (5–10 mice per group). ^†^Humane intervention was required at day 8 for 2 of 10 mice receiving combination treatment. All graphs represent the mean ± SEM. Statistical significance was assessed using unpaired *t* tests (**A** and **B**) and 1-way ANOVA (**D**). *P < 0.05, **P < 0.01, ***P < 0.001.

Our previous work and other studies have shown the combination of DNA-PK inhibitors with topoisomerase II inhibitors (which result in DNA-DSBs) to consistently lead to a large enhancement of cytotoxicity, ranging from 2- to 50-fold (14, 31–33). Here we confirmed that NU5455 can enhance the cytotoxicity of etoposide and doxorubicin, two widely used topoisomerase inhibitors. NU5455 treatment (1 μM, 24 hours) resulted in a 3.5-fold and 4.1-fold enhancement of doxorubicin- and etoposide-induced cytotoxicity (LD_80_) in Huh7 and SJSA-1 cells, respectively (Figure 6A). We found that NU5455 also induced a significant sensitizing effect to doxorubicin in colorectal cancer (HCT116) and HCC (Hep3B and Huh7) cell lines with an LD_80_ sensitization ranging from 3.1- to 5.1-fold, but with no effect in *PRKDC^–/–^* cells, which do not express DNA-PK (Figure 6B and Supplemental Figure 14).

We next sought to examine the effect of NU5455 treatment on parenteral etoposide treatment in the SJSA-1 tumor xenograft model. We examined oral dosing of NU5455 (30 mg/kg) or vehicle immediately prior to etoposide administration (5 mg/kg i.p.) for 5 consecutive days in CD-1 nude mice bearing subcutaneous SJSA-1 tumors. Etoposide itself had a relatively marginal antitumor effect in vivo in this comparatively chemoresistant model, and the dose of NU5455 used did not enhance it significantly (Supplemental Figure 15). We subsequently examined the effect of 100 mg/kg of NU5455 given immediately prior to the administration of etoposide on 5 sequential days (Figure 6C), as this dose can maintain the plasma concentrations of NU5455 for a longer duration in mice (Supplemental Figure 13B). In this experiment, the antitumor activity of etoposide was indeed potentiated significantly; however, it was accompanied by increased weight loss by day 8 (Figure 6D) with a requirement to intervene and humanely euthanize 2 animals because of a sudden deterioration in clinical condition (>15% body-weight loss). These data suggest that it is possible to augment the activity of parenteral etoposide with concomitant DNA-PKcs inhibition, but that this requires a greater dose of NU5455, which, although well tolerated by itself, is associated with greater toxicity in combination and denotes a limited therapeutic gain.

### NU5455 can augment localized doxorubicin chemotherapy in HCC tumor xenografts without increasing toxicity.

**Figure 7:**
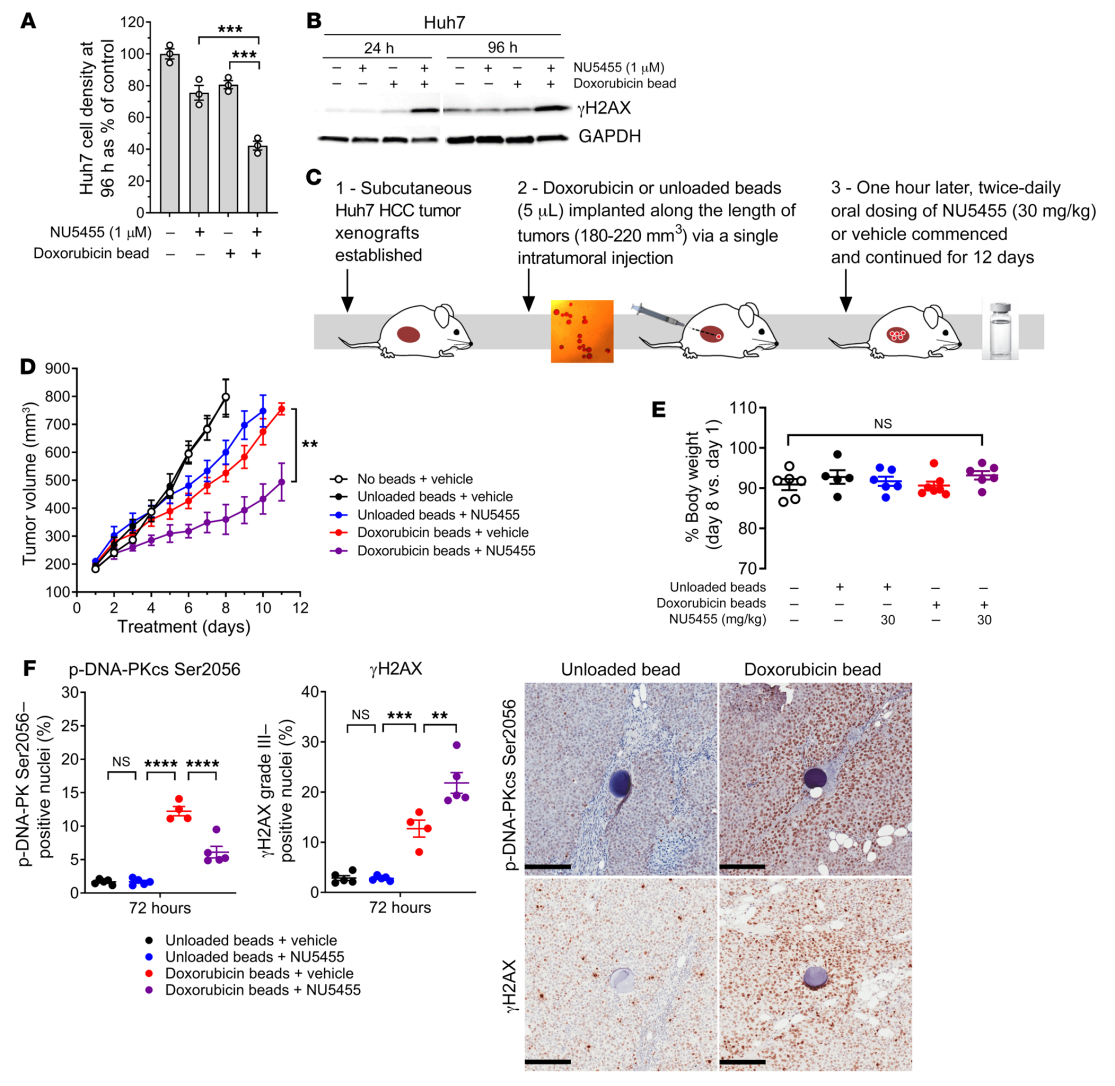
NU5455 augments localized doxorubicin chemotherapy in HCC tumor xenografts without increased toxicity. Exponentially growing Huh7 cells in 6-well plates were treated with NU5455 (1 μM) or DMSO for 1 hour before the addition of a single doxorubicin-loaded 70- to 150-μm DC *M1* bead (25 mg/mL) to the center of each well. (**A** and **B**) Cell density was determined after 96 hours of continuous treatment using sulforhodamine B staining (**A**), and protein extracts were taken for the detection of γH2AX via Western blotting after 24–96 hours of continuous treatment (*n* = 3) (**B**). (**C**) Mice bearing established Huh7 subcutaneous xenografts (180–220 mm^3^) were treated with 5 μL DC *M1* beads loaded with 25 mg/mL doxorubicin, or unloaded beads. Beads were suspended in 30 μL saline and implanted via an intratumoral injection. Twice-daily dosing with NU5455 (30 mg/kg orally, 9-hour dosing interval) or vehicle control was commenced 60 minutes after bead implantation. (**D**) Mean Huh7 tumor volume over time (6 mice per group). (**E**) Percentage body weight at day 8 relative to pretreatment body weight on day 1. (**F**) Percentage phospho–DNA-PKcs Ser2056 nuclear positivity in Huh7 tumor sections, and high (grade III) γH2AX nuclear positivity in a 400-μm radius surrounding individually embedded beads (5 mice per group). Representative images of phospho–DNA-PKcs Ser2056 and γH2AX staining in Huh7 tumor xenografts treated with unloaded or doxorubicin-loaded DC *M1* beads. Scale bars: 200 μm. All graphs represent the mean ± SEM. Statistical significance was assessed using 1-way ANOVA (**A**, **E**, and **F**) and unpaired *t* test (**D**). **P < 0.01, ***P < 0.001, ****P < 0.0001.

Following the observation that concomitant DNA-PK inhibition and systemic chemotherapy may have a narrow therapeutic index, we sought to examine oral NU5455 treatment in combination with localized chemotherapy, and selected doxorubicin-loaded DC *M1* polymer beads (70–150 μm), which are used clinically in the treatment of HCC to deliver transarterial chemoembolization (34). In vitro, continuous incubation with either NU5455 (1 μM) or a single doxorubicin-loaded bead for 96 hours had a relatively small effect (~20% inhibition) on the density of Huh7 cells, which have high DNA-PK expression, whereas combination treatment reduced cell density by 57.7% ± 2.1% (Figure 7A). An analysis of γH2AX protein levels also suggested that NU5455 was able to augment and sustain the DNA damage induced by released doxorubicin in Huh7 cells for at least 96 hours (Figure 7B). Importantly, NU5455 did not directly influence the rate of doxorubicin elution from the beads (Supplemental Figure 16).

Chronic treatment with NU5455 in vivo was then examined in conjunction with localized chemotherapy, by oral administration of NU5455 (30 mg/kg, b.i.d.) to mice bearing subcutaneous HCC xenografts into which doxorubicin-eluting beads had been implanted intratumorally (Figure 7C). An enhanced antitumor effect was observed with combination treatment versus either monotherapy (Figure 7D; mean RTV4 value of 17.8 days for the combination vs. either 10.6 days for NU5455 with unloaded beads or 11.1 days for doxorubicin beads with vehicle, *P* < 0.01 by 2-tailed Mann-Whitney *U* test), and no additional impact on body weight (Figure 7E and Supplemental Figure 17A) or clinical condition was observed versus treatment with vehicle alone. Immunohistochemical analysis after 72 hours of treatment revealed that DNA-PKcs phosphorylation (Ser2056) was elevated in mice treated with doxorubicin-loaded beads, and that this was reduced by NU5455 treatment (Figure 7F). DNA damage (γH2AX) in Huh7 tumors, in the area surrounding doxorubicin-eluting beads, was also significantly augmented by NU5455 treatment at 72 hours. Representative images of stained tumor sections analyzed using a nuclear positivity algorithm are shown (Supplemental Figure 17, B and C). These data indicate that oral administration of NU5455 can enhance the effect of doxorubicin-eluting beads in tumors, without inducing any adverse effects.

## Discussion

Herein we describe the discovery of NU5455, a highly selective orally bioavailable inhibitor of DNA-PKcs. While there has been significant interest in inhibiting DNA-PKcs for therapeutic benefit, we are not aware of any preclinical studies that have critically evaluated both the normal and tumor tissue response to concomitant treatment with a selective DNA-PKcs inhibitor and therapies that induce DNA-DSBs. That inhibition of DNA-PKcs kinase activity can induce radiosensitization of tumor cells has been previously established genetically and pharmacologically (32, 35), and the ability of NU5455 to impart a greater radio-enhancement than small-molecule inhibitors of the DNA repair proteins ATM, PARP, and ATR is consistent with DNA-PK having a critical role in the repair of DNA-DSBs that represent the most lethal DNA damage lesion (1). NU5455 enhanced the effect of IR in vitro across a range of human tumor cell lines, including those that are representative of tumor types commonly treated clinically with external beam radiotherapy (glioblastoma, osteosarcoma, lung and colorectal cancer). However, the magnitude of radio-enhancement observed across the cell panel was unrelated to the intrinsic radiosensitivity of the cell lines or their basal DNA-PK expression (Supplemental Figure 18). This variance in radio-enhancement may relate to a variety of innate cellular differences such as the capacity to overcome oxidative stress, the propensity to resist apoptosis, or, as suggested recently following studies with the DNA-PK inhibitor VX-984 (36), the ability to use compensatory DNA repair mechanisms. It has also been suggested that greater radio-enhancement can be observed in tumor cells following DNA-PKcs inhibition on the basis of p53 status, the loss of functional p53 leading to enhanced cell death through mitotic catastrophe and the induction of apoptosis (37). However, loss of functional p53 through either mutation or deletion (evident in SN40R2, Calu-6, Huh7, Hep3B, U251, 4T1, and HCT116 *TP53^–/–^* cells; Supplemental Table 3) did not explain any differences in radio-enhancement observed with NU5455 in our cell line panel (Figure 2F). Furthermore, we did not find evidence of an enhanced apoptotic response in isogenic HCT116 colorectal tumor cells that lacked *TP53* following treatment with NU5455 and radiation (Supplemental Figure 19). Given that pleiotropic mechanisms may influence the response to combined radiotherapy and DNA-PKcs inhibitor treatment, further studies to elucidate potential determinants of tumor cell sensitivity are warranted. Such work may assist in the development of biomarker strategies to aid the selection of patients with increased sensitivity to combination treatment.

Our data with NU5455 indicates that there is the potential to realize a differential potentiation of radiation-induced DNA damage in tumor versus normal tissues by transient DNA-PKcs inhibition in vivo. This is based on measurements of tumor growth response, acute DNA damage in tumor versus skin or lung, and the lack of an effect of NU5455 treatment on a late normal tissue toxicity (lung fibrosis). Consistent with this, a recent preclinical study examining VX-984 has shown it to increase the efficacy of cranial irradiation against an orthotopically implanted human glioblastoma tumor without an apparent effect on clinical condition (38). Although rodent tissues are known to express less DNA-PK than those of human origin (39), it is unlikely that the differential radiosensitizing effect of NU5455 treatment in human tumors versus normal mouse tissues is attributable to a difference in the murine DNA damage response, given that SCID mice exhibit enhanced skin radiosensitivity (40), and lung fibroblasts from mice with homozygous deletion of the gene encoding DNA-PK (41) or normal murine fibroblasts treated with a DNA-PKcs inhibitor (42) are significantly sensitized to radiation treatment. Furthermore, NU5455 enhanced the cell killing effect of radiation in murine nontumor and tumor cell lines in vitro to a level that was comparable to that observed in most human cell lines. In addition to there being potential cell-intrinsic determinants that may more favorably influence the radio-enhancement of some tumors after DNA-PKcs inhibition, the therapeutic index arising from this pharmacological strategy may be influenced by both the magnitude and the duration of DNA-PKcs inhibition. Certainly, the intrinsic sensitivity of SCID mouse lung to radiation was significantly greater than that of a CD-1 nude mouse lung with NU5455 treatment, suggesting that complete genetic deletion of DNA-PK does not phenocopy transient pharmacological inhibition of DNA-PKcs. Our in vitro data also show the radio-enhancing effect of NU5455 in tumor cells to be highly dependent on the duration of compound incubation following irradiation, which is likely to reflect a time-dependent repair of sublethal damage with extended treatment resulting in the persistence of γH2AX foci. Given that micromolar levels of NU5455 are evident in plasma and tissue for only 3–4 hours in vivo following oral administration of 30 mg/kg, the pharmacokinetics of this compound may conceivably contribute to the observed therapeutic gain. The use of episodic DNA-PK inhibition, as opposed to continuous or sustained inhibition, may also be beneficial in extended combination regimens, since it may avoid a potential effect on telomere maintenance, which has been shown to have DNA-PK dependency in human cells (43). It remains to be determined whether the duration of DNA-PKcs inhibition can result in the differential radio-enhancement of tumors versus normal tissues, but this is also worthy of further examination, and it will be of interest to compare DNA-PK inhibitors with different pharmacokinetic profiles. Indeed, PARP inhibitors have been previously found to augment the toxicity of radiotherapy to the skin and esophagus of mice, which may be influenced by their pharmacokinetic profile or a prolonged pharmacodynamic effect (44, 45).

Our in vitro experiments also indicate that combined IR and NU5455 treatment can have a significantly greater cell-killing effect in proliferating, as opposed to confluent, MCF7 tumor cells. This observation could conceivably provide the basis for a differential sensitivity to IR and NU5455 treatment in proliferating tumor cells versus comparatively quiescent normal tissue. Data generated in MCF10A cells, however, suggest that the impact of cellular proliferation rate on the response to combination treatment can vary between different cell types, potentially reflecting divergences in how different cells process, repair, and respond to DNA-DSBs in the presence of DNA-PK inhibition, which will also be of interest to evaluate further. Alternatively, other facets of tumor pathophysiology, such as tumor hypoxia, may favorably influence the radio-response in vivo, with DNA-PKcs inhibition potentially having a preferential radiosensitizing effect in hypoxic tumor cells (46), which would otherwise represent a more radioresistant population.

When combined with a parenterally administered topoisomerase II inhibitor, NU5455 did show evidence of increased toxicity, suggesting that concomitant use of a DNA-PKcs inhibitor with systemic DNA-DSB therapies is likely to be challenging to implement clinically. Topoisomerase II inhibitors induce DNA damage via catalysis of DNA-DSBs during replication and transcription, which is a more gradual response when compared with the acute DNA-damage effects of IR. Optimal enhancement of such a response is likely to require more extended DNA-PKcs inhibition, perhaps requiring multiple daily dosing or longer half-life compounds, but which may also exacerbate the more widespread insult of the chemotherapy on a range of normal tissues. The concept of combining a DNA-PKcs inhibitor with localized chemotherapy is therefore highly attractive, exemplified by our data that show the efficacy of intratumoral doxorubicin-eluting beads in mice to be enhanced significantly by oral NU5455 administration, without evidence of overt toxicity. This strategy is of potential clinical relevance to the treatment of HCC, in which transarterial chemoembolization (TACE) procedures with doxorubicin-containing beads are used (47) — a disease that has a particularly high mortality and in which new therapeutic interventions have had limited impact (48). Such use may require concomitant administration of a DNA-PK inhibitor for a period of 2–4 weeks, during the period in which doxorubicin is eluted from the beads (49). Since high DNA-PK expression and activation in HCC has been found to correlate with resistance to TACE (14), an HCC patient selection strategy for early clinical evaluation of a DNA-PKcs inhibitor could involve an examination of tumor DNA-PK in a diagnostic biopsy or in circulating tumor cells (50), with those showing highest activity being chosen for combination treatment.

In conclusion, the identification of NU5455 and the preclinical data generated in cancer models suggest that it is possible to achieve an enhanced therapeutic ratio with transient selective pharmacological inhibition of DNA-PKcs in combination with DNA-DSB–inducing therapies. The application of a DNA-PKcs inhibitor, as an adjunct either to radiotherapy or to current HCC treatment, creates exciting therapeutic opportunities that necessitate further evaluation toward clinical implementation.

## Methods

### Reagents.

NU5455 and NU7441 were synthesized by the Newcastle University Medicinal Chemistry Department or NewChem Technologies Ltd. Full synthesis details for NU5455 are provided in patent WO 2010/136778 (example 102; compound 143). NU5455 was dissolved in DMSO for in vitro work, and prepared in *N*-methyl-2-pyrrolidone (NMP)/30% encapsin/PEG400 (1:6:3 vol/vol/vol) or 1% acetic acid (vol/vol) for oral administration in vivo. NU7441 was synthesized as detailed by Leahy et al. (51). KU55933, rucaparib, and VE-821 were purchased from Tocris Bioscience. All other reagents were purchased from Sigma-Aldrich unless otherwise stated. All compounds were dissolved in anhydrous DMSO and stored in small aliquots at –20°C, unless otherwise stated. Doxorubicin solutions were prepared in sterile, molecular-grade water and stored under light-protected conditions at 4°C.

### In vitro kinase selectivity screening.

The in vitro kinase selectivity of NU5455 was determined using the SelectScreen Broad Assay Panel (Thermo Fisher Scientific) involving activity and binding assays (Supplemental Table 1). The ATP concentration used in activity assays was the *K_m_*[app] where available, or 10 or 100 μM as indicated (Supplemental Table 1). Class IA PI3Ks were measured using the p85α subunit.

### Cellular activity of NU5455 against DNA-PK and PI3K.

Exponentially growing MCF7 cells were exposed to a range of concentrations of NU5455 and NU7441 (0.03–10 μM) for 1 hour. To assess the ability of NU5455 and NU7441 to inhibit autophosphorylation of DNA-PKcs at Ser2056, cells were treated with 10 Gy irradiation and incubated for 30 minutes. Alternatively, to investigate the effects of NU5455 and NU7441 against PI3K-dependent phosphorylation of AKT at Ser473, cells were treated with 50 ng/mL insulin-like growth factor-1 (IGF-1; Invitrogen) for 30 minutes. Cell lysates were prepared for the detection of p–DNA-PKcs Ser2056 and total DNA-PKcs, or p–AKT Ser473 and total AKT, respectively, using Western blotting.

### NHEJ plasmid repair assay.

The ability of NU5455 to impair the nonhomologous end joining (NHEJ) ability of cells was assessed using a modified version of a DNA repair assay developed by Nagel et al. (52), which measures the repair of a plasmid encoding blue fluorescent protein (BFP) and GFP linearized by either an ScaI or an AfeI restriction digestion. Full experimental details are given in Supplemental Methods.

### In vivo experiments.

Mice were purchased at 6–10 weeks of age from Charles River UK Ltd. NU5455 was dosed orally at 0.1 mL/10 g body weight using the NMP/30% encapsin/PEG400 formulation for all experimental work with the exception of the HCC xenograft study, in which the 1% (vol/vol) acetic acid formulation was used (Figure 7). All tumor xenograft experiments were conducted in CD-1 nude mice with the exception of the Calu-6 subcutaneous xenograft combination studies (Figure 3), which were performed in BALB/c nude mice. Lung fibrosis experiments were performed in C57BL/6 mice. Comparable NU5455 plasma pharmacokinetic data were obtained following 30 mg/kg oral dosing of NU5455 to each mouse strain examined (Supplemental Figure 13).

### Combining NU5455 and targeted radiotherapy in subcutaneous lung tumor xenografts.

Subcutaneous non–small cell lung cancer xenografts were generated in CD-1 nude or BALB/c nude mice using A549 and Calu-6 cells, respectively, as previously described (53). When tumors reached 100 mm^3^, mice were randomized into 4 groups (*n* = 7–9 per group) to receive (a) vehicle (orally) 30 minutes before mock radiation, (b) NU5455 (30 mg/kg orally) 30 minutes before mock radiation, (c) targeted tumor radiation at a single dose of 3.3 or 10 Gy, or (d) NU5455 (30 mg/kg orally) 30 minutes before 3.3 Gy or 10 Gy targeted tumor radiation. For targeted irradiation of subcutaneous tumors, anesthetized mice were restrained in a lead-shielded container with only the tumor exposed, and x-ray radiation applied using an RS320 irradiation system (Gulmay Medical) at a dose rate of 1.82 Gy/min.

### Combining NU5455 and targeted radiotherapy in orthotopic lung tumor xenografts.

Orthotopic lung xenografts were generated following a published protocol (54) with minor modifications. Briefly, CD-1 nude mice (Charles River Laboratories) were anesthetized with 2% isoflurane and placed in the right lateral decubitus position. Luciferase-expressing Calu-6 cells (1 × 10^6^) in 50 μL of 50% (vol/vol) Matrigel were injected into the left lateral thorax at the lateral dorsal axillary line, 1.5 cm above the lower rib line. The needle was advanced 5 mm into the thorax. After injection, the mouse was turned to the left lateral decubitus position for 5 minutes. Fourteen days after implantation, mice were treated with vehicle, NU5455 (30 mg/kg orally), whole-thorax irradiation (10 Gy), or NU5455 combined with 10 Gy thorax irradiation (*n* = 6–7 per group). For whole-thorax radiation, anesthetized mice were restrained in a lead-shielded container with only the thoracic region exposed. X-ray radiation was applied using an RS320 irradiation system at 2.034 Gy/min (Gulmay Medical). Additional details are presented in Supplemental Methods.

### Combining NU5455 and localized doxorubicin-eluting bead therapy in HCC tumor xenografts.

DC *M1* beads were loaded with doxorubicin at 25 mg/mL as described in Supplemental Methods. Huh7 cells (5 × 10^6^ cells per mouse in 50 μL, 50% vol/vol Matrigel) were implanted s.c. into the right flank of female CD-1 nude mice to generate subcutaneous Huh7 xenografts (180–220 mm^3^). Mice were then treated with 5 μL doxorubicin-loaded or unloaded beads suspended in 30 μL saline administered via an intratumoral injection along the length of each tumor using a 29G needle. One hour after bead implantation, twice-daily dosing with 30 mg/kg NU5455 or vehicle control (1% vol/vol acetic acid in water) was commenced via oral gavage (9-hour dosing interval) and continued for up to 20 days. Tumor volumes were calculated daily using bilateral caliper measurements as described in Supplemental Methods, and body weight measurements taken daily throughout treatment.

### Lung fibrosis studies.

Whole-thorax irradiation (5 Gy) was administered to female C57BL/6 mice (6–8 weeks old) on days 1, 4, 7, and 10 with vehicle or NU5455 treatment. Whole lung sections were stained with Masson’s trichrome to visualize collagen deposition and scored using the modified Ashcroft scale proposed by Hübner et al. (55). Further details are provided in Supplemental Methods.

### Statistics.

Data were analyzed using GraphPad Prism 6.0 software (GraphPad Inc.). Two-tailed, unpaired *t* tests were used to assess statistical significance between 2 treatment groups, unless otherwise specified. ANOVA tests with Bonferroni’s correction for multiple comparisons were used to examine differences between up to 5 treatment groups. Differences in the time taken to reach 4 times the tumor luminescence at the start of treatment (RTL4), and the time taken to reach 2 times the tumor volume at the start of treatment (RTV2) were examined using Kaplan-Meier analyses. *P* values less than 0.05 were considered to indicate statistical significance and are denoted in the figures as follows: *P < 0.05, **P < 0.01, ***P < 0.001, and ****P < 0.0001. NS indicates not significant, *P* > 0.05.

All other methods are detailed in Supplemental Methods. For inquiries concerning the synthesis of NU5455, contact CC, celine.cano@ncl.ac.uk.

### Study approval.

All experiments with mice were approved by the Animal Welfare Ethical Review Boards at Newcastle University (Newcastle upon Tyne, UK) and the University of Oxford (Oxford, UK), in accordance with published guidelines (56) and the UK Animals (Scientific Procedures) Act, 1986, under PPL numbers 70/8769, 60/4222 and 30/3395.

## Author contributions

CEW, YJ, HDT, SK, AW, NP, YZ, SJT, LP, GJ, LML, and JMM performed experiments and acquired data. CEW, YJ, HDT, SK, AW, NP, YZ, SJT, LP, GJ, LML, JMM, EW, CC, DRN, HLR, AJR, and SRW analyzed data. MRVF, PT, RJG, DRN, TR, JP, and CC identified and synthesized NU5455. EW, CC, DRN, HLR, AJR, and SRW conceived, designed, and supervised the research studies. CEW, YJ, EW, AJR, and SRW wrote the manuscript. CEW and YJ share a first-author position for this study, having made equal contributions to the acquisition of data and its analysis and interpretation. CEW also composed the final manuscript figures and therefore appears first in the author list. All authors reviewed and approved the final manuscript.

## Supplementary Material

Supplemental data

## Version 1 10/03/2019

In-Press Preview

## Version 2 11/25/2019

Electronic publication

## Version 3 01/02/2020

Print issue publication
