# Supplementary material for: Selective DNA-PKcs inhibition extends the therapeutic index of localized radiotherapy and chemotherapy
Source: J Clin Invest. 2019 Nov 25;130(1):258–71. doi: 10.1172/JCI127483 (PMC6934184; doi:10.1172/JCI127483)
Supplement: Supplemental data [file jci-130-127483-s144.pdf]

Supplemental Materials

Materials and Methods

Cell lines

Cell lines were obtained from the American Type Culture Collection (ATCC) and maintained in RPMI-1640 medium containing 2 mM L-glutamine and supplemented with 10% (v/v) fetal bovine serum (FBS, Life Technologies), unless otherwise stated. HCT116 parental, DNA-PK-null (*PRKDC* -/-) and p53 null (*TP53* -/-) cells, and HAP-1 parental and DNA-PK-null cells were purchased from Horizon Discovery. MCF10A cells were maintained in Dulbecco's Modified Eagle's Medium: Nutrient Mixture F-12 (DMEM-F12) containing 5% (v/v) horse serum (Life Technologies), 20 ng/ml epidermal growth factor (Peprotech), 0.5 µg/ml hydrocortisone, 100 ng/ml cholera toxin and 10 µg/ml insulin. Huh7 cells were obtained from the Japanese Collection of Research Bioresources (JCRB), and Hep3B, HepG2 and Huh7 cells were maintained in DMEM-F12/HAM medium supplemented with 10% (v/v) FBS and 2 mM L-glutamine. A549, Calu-6 and HAP-1 cell lines were cultured in Advanced DMEM/F12 medium (GIBCO/Life Technologies), supplemented with 5% (v/v) FBS, 2 mM Glutamax, and 50 µg/ml penicillin/streptomycin.

All cell lines were authenticated prior to the commencement of work using short tandem repeat profiling (LGC Standards) and were kept in culture for fewer than 30 passages post-authentication. Cultures were tested for mycoplasma contamination every 3 months using the MycoAlert Mycoplasma Detection Kit (Lonza) and returned negative throughout.

Western blotting

Adhered cells were washed with PBS and scraped into 50-100 µl ice-cold PhosphoSafe extraction reagent (Novagen) containing 4% v/v Complete EDTA-free protease inhibitor cocktail (Roche). Samples were incubated at room temperature for 5 minutes to allow cell lysis, then centrifuged at 16,000 x *g* for 5 minutes at 4°C, and pelleted cell debris discarded. Protein lysates were quantified using the Pierce BCA Protein Assay (ThermoFisher).

For the detection of total and phosphorylated DNA-PK, samples were prepared in 4X XT Sample buffer containing 20X XT Reducing Agent, and run on 3-8% Criterion XT tris-acetate precast gels in 1X XT Tricine running buffer (all BioRad). For the detection of total and phosphorylated AKT and γH2AX, protein extracts were prepared in 4X XT Sample buffer (BioRad) and run on 4-20% Criterion TGX precast gels (BioRad) in 1X tris-glycine running buffer (200 mM glycine, 3.5 mM sodium dodecyl sulphate, 25 mM tris base). All samples were incubated at 95°C for 5 minutes prior to loading.

Proteins separated on tris-acetate gels were transferred onto 0.45 µm PVDF membrane (ThermoFisher) using Tris-Glycine Transfer Buffer 25X (Life Technologies), deionized water and methanol (5% v/v, Fisher Scientific), whereas proteins run on TGX gels were transferred onto 0.45 µm Hybond ECL nitrocellulose blotting membrane (GE). Membranes were blocked using 5% (w/v) milk in tris-buffered saline (15.4 mM Tris-HCl, 137 mM NaCl, pH 7.6) + 0.1% (v/v) Tween 20 (TBST) and incubated with primary antibody solutions prepared in 5% (w/v) milk/TBST or 5% (w/v) bovine serum albumin/TBST overnight at 4°C. Antibodies were used for the detection of: AKT (C67E7, New England Biolabs #4691), pAKT Ser^{473} (193H12, New England Biolabs #4058), DNA-PKcs (43514, Abcam #1832 and BioRad #AHP318), pDNA-PKcs Ser^{2056} (EPR5670, Abcam #124918), GAPDH (0411, Santa Cruz Biotechnology #47724) and γH2AX (20E3, Cell Signaling Technology #9718).

Membranes were then washed in TBST and incubated with HRP-conjugated goat anti-mouse or anti-rabbit antibodies (P0447 and P0448 respectively, Dako) in 5% (w/v) milk/TBST at room temperature for 1-2 hours. Conjugates were detected using Clarity Western ECL Substrate (BioRad) and chemiluminescence imaged using the Fuji LAS-3000 Luminescent Image Analyzer System (Fujifilm).

Densitometry analysis

Densitometry analysis was performed using AIDA Image Analyzer software (Raytest). The intensity of all bands was determined following a background subtraction, and the level of pDNA-PKcs stimulation determined by subtracting basal unstimulated pDNA-PKcs Ser2056 levels. pDNA-PKcs Ser2056 band intensity was calculated relative to total DNA-PKcs band intensity, and is given as percentage expression relative to irradiated, DMSO-treated cells. Alternatively, basal pAKT Ser473 levels were subtracted from all other pAKT bands, and pAKT band intensity calculated relative to total AKT expression and given as a percentage relative to IGF-1 stimulated, DMSO-treated cells.

Non-homologous end-joining plasmid repair assay

pmaxGFP-BFP plasmid preparation

A pmaxBFP plasmid (52) was a gift from Z. Nagel (Massachusetts Institute of Technology). An AseI-AflII fragment containing the BFP expression cassette from pmaxBFP was inserted into the AflII site of a pmaxGFP plasmid (Lonza) by In-Fusion cloning. The pmaxGFP-BFP plasmid was maxi-prepped in *E. coli* DH5α cells under kanamycin selection.

The pmaxGFP-BFP plasmid was linearized, and blunt ends generated via restriction digestion with ScaI or AfeI (New England Biolabs). Control mock-digested and restriction-digested plasmid DNA were separated on a 1% agarose gel prepared using Ultrapure agarose (Invitrogen) and 1X TAE buffer (Invitrogen). Plasmid DNA bands were stained using SYBR Safe DNA gel stain (Invitrogen) and visualized under blue light (460-470 nm excitation, converted from 365 nm UV) using a Visi-Blue Transilluminator (Ultra-Violet Products). The bottom, supercoiled, intact plasmid band, and the digested plasmid band were excised from the gel and purified using a NucleoSpin Gel and PCR Clean-up Kit (Clontech Laboratories) according to the manufacturer's instructions. Purified plasmid DNA was then quantified using a NanoDrop 2000 spectrophotometer (ThermoFisher).

Cell transfection

HEK293T cells were seeded into sterile 96-well plates at high density (Greiner; 20,000 cells/well) and allowed to adhere for 24 hours prior to treatment with 1 µM NU5455, 10 µM KU55933 or DMSO vehicle control. Cells were then transfected with 100 ng linearized or intact plasmid DNA per well using TransIT-LT1 transfection reagent (0.3% v/v, Mirus).

Flow cytometry

24 hours post-transfection, cells were detached from the 96-well plate by the addition of 50 µl 1X Trypsin-EDTA per well and incubation at 37°C for 10 minutes. Cells were re-suspended by gentle pipetting, and incubated for a further 10 minutes before the addition of 200 µl PBS + 5 mM EDTA to promote the formation of a single cell suspension. Cells were transferred into round-bottom 96-well plates (Greiner), and fluorescence analysis performed on an Attune NxT Acoustic Focusing Cytometer (ThermoFisher), using the blue laser with 530/30 emission filter to detect green fluorescence in the BL1 channel, and the

violet laser with 440/50 emission filter to detect blue fluorescence using the VL1 channel. Sample recording was set to 200 µl acquisition volume with sample flow rate 200 µl/minute. Data were analyzed by FCS Express 6 Flow Research Edition (De Novo Software).

Gating strategy

Intact and single-cell populations were defined by gating on the side-scatter and forward-scatter density plots. To define the transfected cell population (single cells expressing one or two fluorescent proteins), a quadrant gate was used with cut off at 1 x 10^3 fluorescent intensity units for both GFP and BFP signals. The non-transfected cell population was excluded by lower left quadrant-to-gate conversion. Representative plots are depicted in Supplemental Figure 3.

Calculation of Relative Fluorescent Expression

Arithmetic mean fluorescent intensity values (total fluorescent intensity of the population divided by the total number of cells) were first normalized to the expression from the undigested plasmid within each treatment group and then to the vehicle control treatment group, as detailed below:

$M_{U\text{-}T}$: Arithmetic mean of fluorescent intensity from expression from undamaged plasmid within treatment group T (DMSO, NU5455 or KU55933)

$M_{D\text{-}T}$: Arithmetic mean of fluorescent intensity from expression from damaged (ScaI or AfeI cut) plasmid within treatment group T

$F_{0\text{-}T}$: fluorescence normalized to undamaged plasmid in treatment group T

$F_{0\text{-}T} = M_{D\text{-}T}/ M_{U\text{-}T}$ within treatment group T

% relative expression = $(F_{0\text{-}T}/ F_{0\text{-}DMSO}) \times 100$

Cell line γH2AX and 53BP1 foci immunofluorescence and quantitation

Cells grown on coverslips or in 96-well plates were treated with 1 or 5 µM NU5455 or DMSO 1 hour prior to irradiation (0, 2.5 or 10 Gy) using an RS 320 X-ray Research System (Gulmay Medical). At set timepoints (15 or 30 minutes, or 1, 4, 5, 6, or 24 hours post-irradiation), cells were washed and fixed in methanol or 3% paraformaldehyde (PFA). Cells were blocked and permeabilized in 1% (v/v) goat serum/1% (w/v) BSA/0.1% (v/v) Triton X100 or 0.1% (v/v) Triton X100 containing 10% (w/v) dried milk powder and 2% (w/v) bovine serum albumin, followed by incubation with antibodies against γH2AX (JBW301, Millipore #05-636) and/or 53BP1 (Cell Signaling #4937). Cells were then washed and incubated with Alexa Fluor 488 goat anti-mouse and/or Alexa Fluor 568 goat anti-rabbit antibodies (A11001 or A11011 respectively, Invitrogen). In A549 and Calu-6, cells were Hoechst stained (5 µg/ml), and the average numbers of γH2AX and 53BP1 foci per cell were quantified from 400 nuclei per sample using an In Cell analyzer 1000 (GE Healthcare). For MCF7 cells, coverslips were mounted using Vectashield mounting medium with DAPI (Vector Labs) and imaged using a Leica TCS SPE confocal fluorescence microscope. Total fluorescence intensity was determined using ImagePro Plus 5.0 software (Media Cybernetics).

Clonogenic survival assays

Exponentially growing cells were pre-treated with 1 µM NU5455 or DMSO vehicle control for 1 h. In the continued presence of NU5455, cells were then incubated with doxorubicin (0.3-1000 nM) or etoposide (0.01 - 10 µM) for 24 hours, or treated acutely with ionizing radiation (0.2-10 Gy) using an RS 320 X-ray Research System (Gulmay Medical) and incubated for a further 24 hours.

Cells were washed in phosphate-buffered saline (PBS), trypsinized and counted. Cells were then reseeded at a known density in drug-free medium into 10 cm dishes (100 - 10 000 cells/dish) and incubated for 8-21 days. Colonies were fixed using Carnoys fixture (75% v/v methanol, 25% v/v acetic acid, Fisher), stained with crystal violet (0.4% w/v in water), and counted using a ColCount automated colony counter (Oxford Optronics). Cell survival was calculated as a percentage of the untreated control.

Alternatively, exponentially growing MCF7 cells were exposed to a range of concentrations of the PARP inhibitor rucaparib, the ATM inhibitor KU55933, the ATR inhibitor VE-821 or NU5455 for 1 hour prior to irradiation with 2 Gy. Cells were then incubated in the continued presence of the DNA repair inhibitors for a further 24 hours prior to replating.

For experiments with confluent populations, cells were seeded in 6-well plates at various densities to determine the appropriate density for confluence. The optimal density was found to be 16 x 10^5/ well (MCF7) or 4 x 10^5/ well (MCF10A), and cells were seeded and grown for 72 hours, at which point they reached confluence. Cells were then pre-treated with 1 µM NU5455 (or DMSO vehicle control) for 1 hour followed by ionizing radiation treatment as described above. The cell cycle phase was confirmed (at 72 hours, prior to treatment) by flow cytometry and microscopy (Supplemental Figure 5).

To examine the time-dependency of NU5455 radio-enhancement, exponentially growing MCF7 cells were pre-treated with 1 µM NU5455 or DMSO vehicle control for 1 hour prior to acute radiation treatment (0.5 - 5 Gy). At set timepoints (1, 2, 4, 6 and 24 hours post-irradiation) NU5455-containing medium was removed from cells, which were then washed with PBS and incubated in drug-free medium until 24 hours post-irradiation, prior to replating as above.

Exponentially growing HAP-1 DNA-PK proficient or deficient cells were seeded into 6-well plates and allowed to re-attach. Cells were then incubated with vehicle (DMSO) or NU5455 (5 µM) 1 hour prior to irradiation and for a further 24 hours post-irradiation. Cells were then washed in PBS and incubated in drug-free medium to allow for colony formation.

EdU staining

To quantify the number of proliferating versus confluent cells, cells were pulsed with 5-ethynyl-2'-deoxyuridine (EdU), and labeled using the Click-iT Plus EdU Alexa Fluor 647 Flow Cytometry Assay Kit together with FxCycle Violet Stain to label DNA (both ThermoFisher) following the manufacturer's instructions. Samples of cells taken from those used in the clonogenic assay were used to confirm the percentage of cells in each cell cycle phase. Samples were acquired on a Symphony A5 flow cytometer (Becton Dickinson, UK).

Cell cycle phase status was also confirmed by microscopy, using cells grown on coverslips in 6-well plates. The Click-iT Plus EdU Alexa Fluor 647 Imaging Kit was used as per the manufacturer's instructions. Coverslips were mounted using Prolong Glass with NucBlue (ThermoFisher) and images were acquired on the Leica TCS SP8 confocal fluorescence microscope.

Proliferation assays

Treated cells were fixed with 10% (w/v) trichloroacetic acid (Fisher) at 4°C overnight. Cells were stained with sulphorhodamine B (SRB, 0.4% (w/v) in water), and cell density quantified

by determining the absorbance at 570 nm of SRB stain dissolved in Tris base (10 mM, pH 10.5) using a spectrophotometer (BMG Labtech).

***In vivo* pharmacokinetic analyses**

All animal experiments were performed in accordance with the Animals (Scientific Procedures) 1986 Act. 8-9 week old mice were purchased from Charles River Laboratories and housed under specific pathogen-free conditions under 12 hour light/dark cycles.

Female CD-1, CD-1 nude, BALB/c nude and C57BL/6 mice were treated with NU5455 (30 or 100 mg/kg prepared in 1% (v/v) acetic acid or NMP:Encapsin (30% aq):PEG4000 (1:6:3 v/v/v) dosed by oral gavage. Blood samples were collected into heparinized tubes at 15, 30, 60, 180 and 360 minutes post-gavage.

Blood samples were centrifuged at 6700 x *g* for 3 minutes, the plasma removed and stored at -20°C prior to analysis for drug by HPLC with fluorescence detection. Aliquots of plasma (50 µl) were vigorously vortexed with acetonitrile (450 µl) and the precipitate removed by centrifugation at 6700 x *g* for 5 minutes. The resulting supernatant (450 µl) was removed and evaporated to dryness under nitrogen. Samples and standards were reconstituted in 90 µl of mobile phase and transferred to HPLC vials.

Samples (20 µl) were applied to a 10 x 0.46 cm Genesis C18 4 µm column (Grace Vydac) fitted with an in-line filter. Compounds were eluted with 0.02 M sodium acetate pH 5:acetonitrile (3:2 v/v) at 1 ml/minute. Analytes were detected by fluorescence at 390 nm following excitation at 242 nm. Plasma concentrations were determined using linear standard curves (0.01 µM – 10 µM, R^2 > 0.98) generated by extracting compounds from human plasma.

Establishment of subcutaneous NSCLC xenograft models

NSCLC subcutaneous xenografts were generated as previously described (53). Briefly, 6-8 week-old female BALB/c nude or CD-1 nude mice (Charles River Laboratories) were anaesthetized with 2% isoflurane (Zoetis), and 5 x 10^6 Calu-6 or A549 cells in 50% Matrigel (BD Biosciences), respectively, were subcutaneously injected into a single site on the back of the mouse. Mouse weights and tumor volumes (1/2 x length x width x depth) were measured three times a week.

Targeted radiotherapy in subcutaneous and orthotopic NSCLC xenograft models

When subcutaneous xenografts reached 100 mm^3, mice were randomized into four groups (n = 7 - 11/group): (i) N-Methyl-2-pyrrolidone (NMP): 30% Encapsin: PEG400 (1:6:3 v/v/v) vehicle *p.o.* 30 minutes prior to mock radiation. (ii) NU5455 (30 mg/kg *p.o.*) 30 minutes prior to mock radiation. (iii) Targeted tumor radiation at a single dose of 3.3 or 10 Gy. (iv) NU5455 (30 mg/kg *p.o.*) 30 minutes prior to 3.3 Gy or 10 Gy targeted tumor radiation. For targeted irradiation of subcutaneous tumors, anaesthetized mice were restrained in a lead-shielded container with only the tumor exposed. X-ray radiation was applied using an RS320 irradiation system (Gulmay Medical) at a dose rate of 1.82 Gy/minute. Three animals from each group were humanely killed at 5 and/or 24 hours after irradiation, and the tumors processed for immunohistochemistry (IHC). The remaining mice were monitored for tumor growth until the sizes of tumors reached 400 mm^3 or up to 38 days after initiation of treatment.

For orthotopic tumor xenografts, 14 days after implantation of Calu-6-luc cells, mice were treated with vehicle, NU5455 (30 mg/kg *p.o.*), whole thorax irradiation (10 Gy), or NU5455

combined with 10 Gy thorax irradiation (*n* = 6 - 7/group). For whole-thorax irradiation, anaesthetized mice were placed in the supine position in a customized rig. The whole thorax was exposed to X-ray radiation generated by the Gulmay Medical RS320 irradiation system (2.034 Gy/minute). The rest of the body was protected by lead shields. Three mice from each treatment group were humanely killed 24 hours after irradiation, and their tumors processed to permit immunohistochemical analysis.

Tumor growth in the remaining mice was monitored by bioluminescent imaging on days 0, 4, 8 and 15 post-treatment. Mice were injected with luciferin (150 mg/kg *i.p.*, Santa Cruz Biotechnology) and bioluminescence images were acquired using the IVIS Spectrum (PerkinElmer) at 5 minute intervals until maximum light was collected. Imaging commenced 5 minutes post-luciferin injection, and mice were anaesthetized with isoflurane during image acquisition. Data were analyzed using LivingImage software (PerkinElmer).

Normal lung tissue toxicity studies

In the above study, normal lung tissue from CD-1 nude mice bearing orthotopic Calu-6 xenografts was also harvested 24 hours post-treatment with NU5455 ± 10 Gy IR. Additionally, DNA-PK-defective severe combined immunodeficient (SCID) mice were treated with 10 Gy thoracic radiation and humanely killed 24 hours post-irradiation. Lung tissues were then processed for immunohistochemical analysis as detailed below.

In a subsequent study, female C57BL/6 mice (6-8 weeks old) were randomly assigned to 4 treatment groups: Group 1: no treatment; Group 2 : 5 Gy whole-thorax irradiation (as detailed above) on days 1, 4, 7 and 10; Group 3: vehicle (NMP:30% Encapsin:PEG400 1:6:3 v/v/v) *p.o.* 30 minutes before and 5 hours after each dose of 5 Gy whole-thorax irradiation

on days 1, 4, 7 and 10; Group 4: 30 mg/kg NU5455 *p.o.* 30 minutes before and 5 hours after each dose of 5 Gy whole-thorax irradiation on days 1, 4, 7 and 10 (n = 5 - 9/group). Mice were weighed twice a week, and humanely killed after 24 weeks with lungs being taken for fibrosis scoring.

Immunohistochemistry in lung tumor xenografts and normal lung tissue

Tissues were fixed in 4% paraformaldehyde (PFA) and paraffin-embedded. Sections were stained using primary antibodies against γH2AX (JBW301, 1:1000, Millipore #05-636), 53BP1 (1:500, Cell Signaling #4937), and cleaved caspase 3 (CC3, 1:600, Cell Signaling #9661) and the EnVision G2 Doublestain System (Dako #K5361), according to the manufacturer's instructions. One tissue section from each of 3 mice per group was examined, and the whole section (excluding necrotic areas) analyzed using ImageScope analysis software (Aperio Technologies). γH2AX and 53BP1 foci were manually counted in 200 nuclei from each tumor sample. The percentage of γH2AX and 53BP1 positive nuclei in lung epithelial cells was quantified manually in normal lung samples across 5 randomly-chosen fields of view per sample.

Fibrosis scoring

Masson's trichrome staining was used to evaluate fibrosis in lung samples. Slides were deparaffinized and stained using a Masson's trichrome staining kit (Sigma) according to the manufacturer's instructions. Slides were then dehydrated, mounted and scanned using the Aperio ScanScope CS digital slide scanner (x20 magnification).

Scoring of lung samples stained by Masson's trichrome was performed and scored following the modified Ashcroft scale proposed by Hübner *et al.* (55). Samples were anonymized prior to scoring and a single central lung slice evaluated per animal.

Combining systemic administration of etoposide + NU5455 *in vivo*

SJSA1 cells (1 x 10^7 cells/mouse in 50 µl, 50% v/v Matrigel) were implanted *s.c.* into the right flank of female CD-1 nude mice. When tumor volumes reached 103-121 mm^3 mice were treated daily, for five consecutive days, with NU5455 (30 mg/kg or 100 mg/kg once-daily *p.o.* in NMP:Encapsin (30% aq):PEG4000 (1:6:3 v/v/v)) or vehicle, followed immediately with etoposide (5 mg/kg *i.p.* in saline). Body weight and tumor volume measurements were taken daily during treatment and three times per week post-treatment. Tumor volume was calculated from two-dimensional electronic caliper measurements (Mitutoyo) using the equation 'width^2 x length/2', where width was the smaller side, and length the longer dimension of the tumor. Mice were humanely killed when; (i) an adverse effect on clinical condition was evident, (ii) tumor bilateral measurements reached 10 mm x 10 mm, (iii) a tumor measurement in any dimension reached 15 mm, or (iv) at study termination.

Doxorubicin-eluting DC *M1* beads *in vitro*

DC *M1* 70-150 µm polyvinyl alcohol beads were purchased from Biocompatibles. Excess saline solution was removed from DC *M1* beads using an 18G blunt-fill filter needle (5 µm filter, Beckton-Dickinson), and beads were loaded with doxorubicin at 25 mg/ml under light-protected conditions, overnight at 4°C.

Doxorubicin-loaded beads (25 mg/ml) suspended in molecular-grade water were added to wells of a 96-well plate containing sterile DI water, PBS or phenol red-free DMEM cell culture medium + 2 mM sterile-filtered L-glutamine. Elution of doxorubicin in the presence of NU5455 (10 µM) or DMSO vehicle control compared to untreated solutions was assessed. The fluorescence of the elution medium in each well was measured using a FLUOstar Omega Microplate Reader (BMG Labtech), excitation λ: 485-520 nm, emission λ: 580-610 nm. Readings were taken every 2 hours for the first 10 hours and then at 24 hour intervals. Plates were incubated under light-protected conditions at 37°C between readings. Doxorubicin concentrations were quantified against a free doxorubicin standard curve (0 - 5000 ng/ml), and elution calculated as percentage of total elution over time.

Exponentially growing Huh7 cells seeded in 6-well plates (Corning) were pre-treated with 1 µM NU5455 or vehicle control for 1 hour. A single doxorubicin-loaded DC *M1* bead suspended in sterile, molecular-grade water, or an unloaded bead, was added to the center of each well. Successful addition of a single bead per well was verified visually down a microscope. Cells were incubated for a further 24-96 hours before protein lysates were generated for Western blotting, and wells fixed for SRB staining.

γH2AX immunohistochemistry in HCC tumor xenografts

FFPE tumor sections (4 µm) were dewaxed in xylene and hydrated in descending concentrations of ethanol. Endogenous tissue peroxidase activity was quenched using 3% v/v hydrogen peroxide. Antigen retrieval was performed using 1 mM EDTA, pH 8.0 in a microwave (Kenwood). Tissue sections were blocked for avidin and biotin using the

Avidin/Biotin Blocking Kit (Vector Laboratories), and washed in TBST between all subsequent steps. Slides were blocked in 20% v/v pig serum in DI water, and sections incubated with an antibody against γH2AX (20E3, Cell Signaling Technology #9718, 1 in 200 in TBST) in a humidified container at 4°C overnight.

Sections were then incubated with polyclonal swine anti-rabbit IgG, biotinylated (Dako #E0353, 1 in 200 in TBST), followed by VectaStain RTU Elite ABC reagent (Vector Laboratories). Slides were stained using DAB Peroxidase Substrate Kit (Vector Laboratories) prepared according to the manufacturer's instructions. Slides were washed in DI water, then counterstained in filtered haematoxylin solution. Background staining was minimized by exposure to acid alcohol solution (1% v/v hydrochloric acid in 70% v/v ethanol, Fisher), and staining blued by incubation in Scott's tap water. Stained slides were then dehydrated in ascending concentrations of ethanol, followed by clearing in xylene. Glass coverslips (Dako) were mounted onto slides using DPX mounting medium.

pDNA-PKcs Ser^{2056} immunohistochemistry in HCC tumor xenografts

Tumor sections were dewaxed in xylene, hydrated, and endogenous peroxidase activity quenched as detailed above. Antigen retrieval was performed in 1X citrate buffer (10 mM citric acid (Acros), pH 6.0 + 0.5% v/v Tween 20), using a decloaker (A. Menarini Diagnostics). Tissue sections were blocked in 1% w/v BSA and 1% v/v normal goat serum in PBS (1 hour, RT). Sections were then incubated at 4°C overnight in a humidified container with an antibody against pDNA-PKcs Ser2056 (Abcam #18192, 1:1000 in 1% w/v BSA and 1% v/v normal goat serum).

Sections were washed with TBST before incubation with EnVision+ anti-rabbit Polymer-HRP solution (Dako #K4002). Slides were washed in TBST, and antibody conjugates visualized by incubation with ImmPACT DAB peroxidase substrate working solution. Staining was quenched by washing with DI water, and slides counterstained, dehydrated and mounted as above.

Digital IHC analysis

IHC slides were digitized via conversion using the Aperio ScanScope CS Brightfield scanner (Leica Biosystems) at x20 zoom. Percentage hepatocyte nuclear positivity for pDNA-PKcs Ser2056 and γH2AX was determined using a nuclear algorithm generated with the Aperio ImageScope software v11.2.0.780 (Leica Biosystems). The algorithm was adapted from the Nuclear v9 algorithm with the following parameter adjustments: Area - 50-800 μm^2, Roundness - 0.2, Elongation - 0.2. The algorithm assigned nuclei an intensity value between 0 and 255, where 255 indicated completely negative staining and 0 was completely positive. A cytoplasmic intensity threshold of 230 was applied to correct for background DAB staining, and positive nuclei were graded according to stain intensity, with Grade I nuclei being weakly positive (210-189), Grade II nuclei indicating medium intensity staining (188-163), and Grade III indicating strong nuclear intensity (≤162).

γH2AX positivity analysis was performed on areas of 500 000 μm^2 ± 5% around individual doxorubicin-loaded DC *M1* beads, and at least three beads were analyzed from each of five mice per treatment group. Data are presented as percentage positive nuclei, with scoring presented as low (Grade I), medium (Grade II) and high (Grade III) nuclear positivity. pDNA-

PKcs Ser2056 positive nuclei were assessed across whole tumor sections containing individually embedded beads.

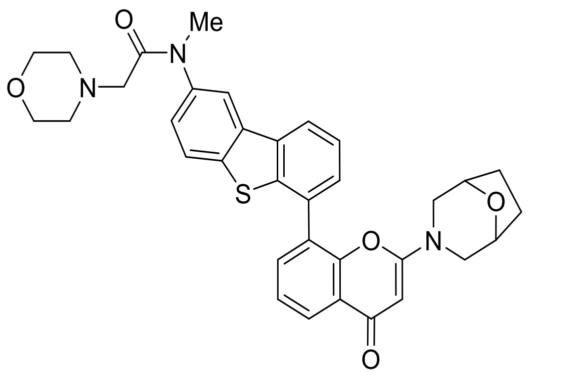

NU5455

N-(6-(2-(8-oxa-3-azabicyclo[3.2.1]octan-3-yl)-4-oxo-4*H*-chromen-8-yl)dibenzo[*b*,*d*]thiophen-2-yl)-*N*-methyl-2-morpholinoacetamide

λ_{max} (EtOH/nm) 214.6, 242.4, 291.4

IR (neat) ν_{max}/cm^{-1} 3049, 2953 (CH), 2853, 1655 (C=O), 1619 (C=O), 1562 (C=C)

^{1}H NMR (500 MHz, $CDCl_3$) δ 1.57 (2H, bs, 2 x C*H*$_2$-HAT), 1.75 (2H, bs, 2 x C*H*$_2$-HAT), 2.40 (4H, bs, 2 x CH_2-N-Morph.), 2.88–3.03 (6H, m, 2 x CH_2-N-HAT and CH_2), 3.32 (3H, bs, CH_3), 3.62 (4H, bs, CH_2-O-Morph.), 4.13 (2H, bs, 2 x CH-HAT), 5.38 (1H, s, CH), 7.20 (1H, dd, *J* = 2.1 and 8.6 Hz, H-Ar), 7.42 (1H, ap. t, *J* = 7.8 Hz, H-Ar), 7.49 (1H, d, *J* = 7.4 Hz, H-Ar), 7.57 (1H, ap. t, *J* = 7.4 Hz, H-Ar), 7.70 (1H, dd, *J* = 1.5 and 7.4 Hz, H-Ar), 7.79 (1H, d, *J* = 8.8 Hz, H-Ar), 8.00 (1H, d, *J* = 1.8 Hz, H-Ar), 8.12 (1H, d, *J* = 7.8 Hz, H-Ar), 8.21 (1H, dd, *J* = 1.7 and 7.9 Hz, H-Ar)

^{13}C NMR (125 MHz, $CDCl_3$) δ 27.5 (CH_2-HAT), 37.9 (CH_3), 49.8 (CH_2-N-HAT), 50.8 (CH_2-Morph.), 53.7 (CH_2-N-Morph.), 60.1 (CH_2-amide), 66.8 (CH_2-O-Morph.), 72.8 (CH-HAT), 86.76 (CH) 120.7 (C-Ar), 121.5 (C-Ar), 123.7 (C-Ar), 123.9 (C-Ar), 124.9 (C-Ar), 125.0 (C-Ar), 126.2 (C-Ar), 128.1 (C-Ar), 128.8 (C-Ar), 131.6 (C-Ar), 133.1 (C-Ar), 135.3 (C-Ar), 136.7 (C-Ar), 138.7 (C-Ar), 140.5 (C-Ar), 140.8 (C-Ar), 150.5 (C-Ar), 163.2 (C=O), 169.2 (C-N-HAT), 176.7 (C=O)

HRMS calculated for $C_{15}H_{15}NO_4$ *m/z* 596.2214 $[M+H]^+$, found 596.2210

HPLC purity >95%

Supplemental Figure 1. Chemical structure and analytical characterisation of NU5455. Fourier transform infrared (FTIR) spectra were measured using an Agilent Cary 630 FTIR spectrometer on a neat sample. UV spectra were recorded in ethanol on a Hitachi U-2800A spectrophotometer. High resolution mass spectrometry (HRMS) was provided by the ESPRC National Mass Spectrometry Service, University of Wales, Swansea. Analytical purity was determined using a Waters XTerra RP18, 5 µm (4.6 × 150 mm) column at 1 ml per minute using 0.1% aq. HCOOH and MeCN with a gradient of 5-100% over 15 minutes. ^{1}H NMR spectra were obtained using a Bruker Avance III 500 spectrometer at a frequency of 500 MHz. ^{13}C NMR spectra were acquired using the Bruker Avance III 500 spectrometer operating at a frequency of 125 MHz.

Kinase	[ATP]	%	Assay
DNA-PK	Km app	98.3	Z
PIK3C3 (hVPS34)	Km app	85.9	A
PIK3CD/PIK3R1 (p110δ/p85α)	Km app	57.5	A
PIK3C2B (PI3K-C2 beta)	10	28.9	A
SNF1LK2	Km app	26.6	Z
BRAF V599E	100	18.3	Z
WEE1	n/a	18.2	L
HCK	Km app	18.0	Z
NEK2	Km app	17.8	Z
ALK	Km app	17.3	Z
FGFR3 K650M	n/a	16.5	L
STK39 (STLK3)	n/a	16.2	L
SIK3	n/a	16.1	L
ACVR2B	n/a	15.7	L
LYN A	Km app	15.2	Z
BMPR1B (ALK6)	n/a	14.5	L
INSRR (IRR)	Km app	14.1	Z
PLK2	Km app	14.1	Z
PRKACA (PKA)	Km app	14.0	Z
EIF2AK2 (PKR)	n/a	13.3	L
NEK1	Km app	12.9	Z
ACVR1 (ALK2) R206H	n/a	12.6	L
PRKX	Km app	12.5	Z
FGR	Km app	12.1	Z
MAPK8 (JNK1)	100	12.0	Z
CLK1	Km app	11.7	Z
MARK3	Km app	11.4	Z
RET V804M	n/a	11.3	L
PDGFRB (PDGFR beta)	Km app	11.0	Z
RPS6KA2 (RSK3)	Km app	10.8	Z
PRKCN (PKD3)	Km app	10.7	Z
STK16 (PKL12)	n/a	10.5	L
FES (FPS)	Km app	10.4	Z
ABL1	Km app	10.4	Z
MUSK	Km app	10.3	Z
MAPK1 (ERK2)	Km app	10.1	Z
BRAF	100	9.9	Z
IGF1R	Km app	9.7	Z
MAPK14 (p38 alpha)	Km app	9.7	Z
LCK	Km app	9.6	Z
MAPK11 (p38 beta)	Km app	9.5	Z
MAPK3 (ERK1)	Km app	9.5	Z
SRC	Km app	9.5	Z
CHEK1 (CHK1)	Km app	9.3	Z
CAMK2D (CaMKII delta)	Km app	9.3	Z
PLK1	Km app	9.2	Z
RET Y791F	Km app	9.2	Z
FLT3	Km app	9.1	Z
MAPK10 (JNK3)	100	9.0	Z
CDK9 (Inactive)	n/a	9.0	L
MAP2K1 (MEK1)	100	8.8	Z
CSNK2A2 (CK2 alpha 2)	Km app	8.7	Z
SGK (SGK1)	Km app	8.5	Z
MERTK (cMER)	Km app	8.3	Z
FYN	Km app	8.3	Z
TEC	n/a	8.1	L
KIT V654A	n/a	8.1	L
ROS1	Km app	8.0	Z
FGFR3 K650E	Km app	7.9	Z
RAF1 (cRAF) Y340D Y341D	100	7.9	Z
PI4KA (PI4K alpha)	10	7.8	A
HIPK2	Km app	7.8	Z
CSK	Km app	7.6	Z
PRKCG (PKC gamma)	Km app	7.6	Z
SRMS (Srm)	Km app	7.5	Z
YES1	Km app	7.4	Z
RPS6KA5 (MSK1)	Km app	7.4	Z
TNIK	n/a	7.3	L
CSNK2A1 (CK2 alpha 1)	Km app	7.1	Z
FER	Km app	7.1	Z
CDK16 (PCTK1)/cyclin Y	n/a	7.0	L
MST4	Km app	6.9	Z
FLT1 (VEGFR1)	Km app	6.5	Z
DYRK1A	Km app	6.4	Z
PRKCD (PKC delta)	Km app	6.2	Z
EGFR (ErbB1)	Km app	6.0	Z
CDC7/DBF4	n/a	6.0	L
JAK3	Km app	6.0	Z
ABL1 Y253F	Km app	5.9	Z
EGFR (ErbB1) d746-750	n/a	5.8	L
MAPK13 (p38 delta)	Km app	5.8	Z
PAK7 (KIAA1264)	Km app	5.7	Z
FRAP1 (mTOR)	Km app	5.7	Z
RPS6KA1 (RSK1)	Km app	5.7	Z
KIT Y823D	n/a	5.6	L
ERBB2 (HER2)	Km app	5.6	Z
ITK	Km app	5.5	Z
KIT	Km app	5.3	Z
CDK7/cyclin H/MNAT1	Km app	5.3	A
RET	Km app	5.2	Z
EPHA5	Km app	5.1	Z
FGFR1 V561M	n/a	5.1	L
PTK2 (FAK)	Km app	5.1	Z
EPHB1	Km app	5.1	Z
CDK14 (PFTK1)/cyclin Y	n/a	5.1	L
MARK1 (MARK)	Km app	5.1	Z
NTRK2 (TRKB)	Km app	5.1	Z
CDC42 BPA (MRCKA)	Km app	5.0	Z
DYRK2	n/a	5.0	L
EPHA2	Km app	4.8	Z
STK33	n/a	4.7	L

Kinase	[ATP]	%	Assay
LATS2	n/a	4.7	L
PRKCZ (PKC zeta)	Km app	4.7	Z
NLK	n/a	4.7	L
FGFR1	Km app	4.7	Z
MINK1	Km app	4.6	Z
ULK3	n/a	4.5	L
BMX	Km app	4.4	Z
CSF1R (FMS)	Km app	4.3	Z
CSNK1G3 (CK1 gamma 3)	Km app	4.2	Z
STK32C (YANK3)	n/a	4.2	L
STK22D (TSSK1)	Km app	4.2	Z
PIK3CA/PIK3R1 (p110α/p85α)	Km app	4.2	A
TAOK3 (JIK)	n/a	4.2	L
TYK2	Km app	4.2	Z
RPS6KA4 (MSK2)	Km app	4.2	Z
PTK2B (FAK2)	Km app	4.2	Z
CLK2	Km app	4.1	Z
STK24 (MST3)	Km app	4.1	Z
CAMK1D (CaMKI delta)	Km app	4.1	Z
INSR	Km app	4.1	Z
MAP3K11 (MLK3)	n/a	4.0	L
PAK2 (PAK65)	Km app	4.0	Z
IRAK1	Km app	4.0	A
SRC N1	Km app	3.9	Z
MAP2K2 (MEK2)	100	3.9	Z
SRPK2	Km app	3.9	Z
RPS6KA6 (RSK4)	Km app	3.8	Z
TAOK1	n/a	3.7	L
GRK6	Km app	3.7	Z
MAPK15 (ERK7)	n/a	3.6	L
PKN1 (PRK1)	Km app	3.6	Z
MAP3K8 (COT)	100	3.5	Z
FLT4 (VEGFR3)	Km app	3.5	Z
GRK4	Km app	3.5	Z
FRK (PTK5)	Km app	3.4	Z
FYN A	n/a	3.3	L
CDK1/cyclin A2	n/a	3.3	L
KIT V559D T670I	n/a	3.1	L
SPHK2	10	3.1	A
FGFR3 G697C	n/a	3.1	L
RIPK2	n/a	3.1	L
LRRK2 G2019S	Km app	3.0	A
PAK6	Km app	3.0	Z
LYN B	Km app	3.0	Z
DAPK2	n/a	2.9	L
EPHA4	Km app	2.9	Z
PIK3CG (p110 gamma)	Km app	2.9	A
HIPK1 (Myak)	Km app	2.8	Z
ADRBK2 (GRK3)	Km app	2.8	Z
MAPK9 (JNK2)	100	2.8	Z
STK22B (TSSK2)	Km app	2.8	Z
STK17B (DRAK2)	n/a	2.7	L
MAP4K2 (GCK)	Km app	2.7	Z
CDK2/cyclin A	Km app	2.7	Z
EPHA1	Km app	2.7	Z
CDK2/cyclin E1	n/a	2.7	L
TESK2	n/a	2.7	L
SPHK1	Km app	2.7	A
AMPK A2/B1/G1	Km app	2.6	Z
DYRK3	Km app	2.6	Z
CSNK1D (CK1 delta)	Km app	2.6	Z
ACVR2A	n/a	2.5	L
DDR2 T654M	n/a	2.5	L
JAK2	Km app	2.5	Z
HIPK3 (YAK1)	Km app	2.5	Z
AMPK (A1/B1/G2)	n/a	2.4	L
MYO3B (MYO3 beta)	n/a	2.3	L
PASK	Km app	2.3	Z
MAP3K5 (ASK1)	n/a	2.3	L
ACVRL1 (ALK1)	n/a	2.3	L
EGFR (ErbB1) L861Q	Km app	2.2	Z
RPS6KA3 (RSK2)	Km app	2.1	Z
STK23 (MSSK1)	Km app	2.0	Z
ALK R1275Q	n/a	2.0	L
TBK1	Km app	2.0	Z
ACVR1B (ALK4)	Km app	2.0	Z
CDK3/cyclin E1	n/a	1.9	L
KDR (VEGFR2)	Km app	1.8	Z
MAP2K6 (MKK6)	100	1.8	Z
TNK2 (ACK)	n/a	1.8	L
ULK1	n/a	1.8	L
LRRK2 R1441C	Km app	1.8	A
AURKB (Aurora B)	Km app	1.7	Z
CDK11 (Inactive)	n/a	1.7	L
LRRK2	Km app	1.7	A
ALK F1174L	n/a	1.6	L
MAP2K3 (MEK3)	n/a	1.6	L
DAPK1	Km app	1.6	A
EPHA6	n/a	1.6	L
MAP4K1 (HPK1)	n/a	1.6	L
CDK1/cyclin B	Km app	1.6	Z
SLK	n/a	1.5	L
MAP3K10 (MLK2)	n/a	1.5	L
DCAMKL2 (DCK2)	Km app	1.5	Z
MLCK (MLCK2)	n/a	1.5	L
MAPKAPK3	Km app	1.4	Z
CLK4	n/a	1.4	L
LIMK2	n/a	1.4	L
NTRK1 (TRKA)	Km app	1.3	Z
PIK3C2A (PI3K-C2 alpha)	Km app	1.3	A
SIK1	n/a	1.3	L

Kinase	[ATP]	%	Assay
KIT D820E	n/a	1.3	L
GRK1	n/a	1.3	L
EPHA7	n/a	1.2	L
BRSK2	n/a	1.2	L
NUAK1 (ARK5)	Km app	1.2	A
EPHB3	Km app	1.1	Z
ABL1 Q252H	n/a	1.1	L
CDK9/cyclin K	n/a	1.0	L
NUAK2	n/a	1.0	L
CDK2/cyclin O	n/a	0.9	L
CAMKK2 (CaMKK beta)	n/a	0.9	L
AMPK (A1/B2/G1)	n/a	0.9	L
FGFR3	Km app	0.9	Z
HIPK4	Km app	0.9	Z
ULK2	n/a	0.9	L
KIT D816V	n/a	0.9	L
CAMK1 (CaMK1)	10	0.8	A
PTK6 (Brk)	Km app	0.7	Z
CAMK2B (CaMKII beta)	Km app	0.7	Z
ACVR1 (ALK2)	n/a	0.7	L
KIT A829P	n/a	0.7	L
DDR2	n/a	0.7	L
ABL1 H396P	n/a	0.6	L
MERTK (cMER) A708S	n/a	0.6	L
ALK C1156Y	n/a	0.6	L
MAPKAPK5 (PRAK)	Km app	0.6	Z
AKT1 (PKB alpha)	Km app	0.5	Z
DDR2 N456S	n/a	0.5	L
NTRK3 (TRKC)	Km app	0.4	Z
DYRK4	Km app	0.3	Z
ZAP70	Km app	0.3	Z
CSNK1A1 (CK1 alpha 1)	Km app	0.3	Z
MYLK (MLCK)	n/a	0.2	L
MKNK2 (MNK2)	n/a	0.2	L
AMPK (A1/B1/G3)	n/a	0.2	L
MAP3K2 (MEKK2)	n/a	0.1	L
CDK8/cyclin C	n/a	0.1	L
MARK2	Km app	0.1	Z
ICK	n/a	0.0	L
FGFR2	Km app	0.0	Z
AKT2 (PKB beta)	Km app	0.0	Z
MAP2K1 (MEK1) S218D S222D	n/a	-0.1	L
EPHB2	Km app	-0.1	Z
TGFBR2	n/a	-0.1	L
TEK (Tie2)	Km app	-0.1	Z
AURKA (Aurora A)	Km app	-0.1	Z
KIT D816H	n/a	-0.2	L
KIT N822K	n/a	-0.2	L
PRKACG (PRKAC gamma)	n/a	-0.2	L
SYK	Km app	-0.2	Z
EPHA3	n/a	-0.3	L
TEK (TIE2) Y1108F	n/a	-0.3	L
TLK2	n/a	-0.3	L
TGFBR1 (ALK5)	n/a	-0.4	L
CLK3	Km app	-0.5	Z
MAP3K14 (NIK)	n/a	-0.5	L
AKT3 (PKB gamma)	Km app	-0.5	Z
MAP3K9 (MLK1)	Km app	-0.5	Z
IKBKB (IKK beta)	Km app	-0.6	Z
CAMK2A (CaMKII alpha)	Km app	-0.6	Z
PRKCB1 (PKC beta I)	Km app	-0.7	Z
TTK	n/a	-0.7	L
ZAK	n/a	-0.7	L
IKBKE (IKK epsilon)	Km app	-0.7	Z
CAMK2G (CaMKII gamma)	n/a	-0.8	L
TLK1	n/a	-0.8	L
MAPK12 (p38 gamma)	Km app	-0.8	Z
PRKCI (PKC iota)	Km app	-0.8	Z
ABL1 M351T	n/a	-1.0	L
PKN2 (PRK2)	n/a	-1.0	L
PIM1	Km app	-1.0	Z
CDK5/p25	Km app	-1.1	Z
SRPK1	Km app	-1.1	Z
BRSK1 (SAD1)	Km app	-1.1	Z
MAP3K3 (MEKK3)	n/a	-1.1	L
AMPK A1/B1/G1	Km app	-1.2	Z
WNK3	n/a	-1.2	L
LATS1	n/a	-1.3	L
STK32B (YANK2)	n/a	-1.4	L
CDK9/cyclin T1	Km app	-1.4	A
DMPK	n/a	-1.5	L
MET D1228H	n/a	-1.5	L
CDK2/cyclin A1	n/a	-1.6	L
ALK L1196M	n/a	-1.7	L
LIMK1	n/a	-1.7	L
RET G691S	n/a	-1.8	L
DYRK1B	Km app	-1.8	Z
DDR1	n/a	-1.8	L
BTK	Km app	-2.0	Z
ADRBK1 (GRK2)	Km app	-2.0	Z
PRKCA (PKC alpha)	Km app	-2.0	Z
MARK4	Km app	-2.0	Z
MAP4K5 (KHS1)	Km app	-2.0	Z
MAP4K3 (GLK)	n/a	-2.1	L
CDK5 (Inactive)	n/a	-2.1	L
NEK7	Km app	-2.1	Z
MYLK2 (skMLCK)	Km app	-2.2	Z
RIPK3	n/a	-2.3	L
PDGFRA V561D	Km app	-2.3	Z
AMPK (A2/B2/G1)	n/a	-2.3	L
EEF2K	Km app	-2.4	Z

Kinase	[ATP]	%	Assay
GSK3B (GSK3 beta)	Km app	-2.5	Z
BMPR2	n/a	-2.5	L
WNK2	n/a	-2.5	L
CDC42 BPB (MRCKB)	Km app	-2.5	Z
AMPK (A2/B2/G2)	n/a	-2.6	L
SGK2	Km app	-2.7	Z
AXL R499C	n/a	-2.7	L
PRKG1	Km app	-2.7	Z
KIT T670E	n/a	-2.8	L
ERBB4 (HER4)	Km app	-2.8	Z
PLK4	n/a	-2.8	L
TXK	Km app	-2.9	Z
GRK7	Km app	-3.0	Z
ABL1 T315I	Km app	-3.0	Z
MAPKAPK2	Km app	-3.1	Z
FGFR4	Km app	-3.1	Z
CASK	n/a	-3.1	L
GRK5	Km app	-3.2	Z
EGFR (ErbB1) T790M	Km app	-3.3	Z
PAK4	Km app	-3.3	Z
TEK (TIE2) R849W	n/a	-3.3	L
MAP3K7/MAP3K7IP1 (TAK1-TAB1)	n/a	-3.4	L
DAPK3 (ZIPK)	Km app	-3.4	Z
RET M918T	n/a	-3.5	L
PHKG2	Km app	-3.6	Z
PRKCQ (PKC theta)	Km app	-3.7	Z
CAMKK1 (CAMKKA)	n/a	-3.8	L
PIK3CB/PIK3R1 (p110β/p85α)	Km app	-3.8	A
FLT3 D835Y	Km app	-3.9	Z
LRRK2 I2020T	Km app	-4.0	A
ROCK2	Km app	-4.1	Z
CHUK (IKK alpha)	Km app	-4.2	A
AXL	Km app	-4.2	Z
PIM2	Km app	-4.3	Z
PAK1	Km app	-4.4	Z
BMPR1A (ALK3)	n/a	-4.4	L
STK4 (MST1)	Km app	-4.4	Z
PRKCH (PKC eta)	Km app	-4.6	Z
SGKL (SGK3)	Km app	-4.6	Z
PI4KB (PI4K beta)	Km app	-4.7	A
PRKCE (PKC epsilon)	Km app	-4.9	Z
MAP2K6 (MKK6) S207E T211E	n/a	-4.9	L
PHKG1	Km app	-5.0	Z
PDGFRA D842V	Km app	-5.0	Z
PRKG2 (PKG2)	Km app	-5.1	Z
TYRO3 (RSE)	Km app	-5.1	Z
TAOK2 (TAO1)	Km app	-5.1	Z
EPHB4	Km app	-5.3	Z
AURKC (Aurora C)	Km app	-5.4	Z
GSK3A (GSK3 alpha)	Km app	-5.5	Z
MAP4K4 (HGK)	Km app	-5.6	Z
JAK1	Km app	-5.7	Z
STK38 (NDR)	n/a	-5.9	L
MATK (HYL)	Km app	-6.3	Z
ABL1 E255K	Km app	-6.6	Z
STK17A (DRAK1)	n/a	-6.6	L
JAK2 JH1 JH2	Km app	-6.7	Z
PRKACB (PRKAC beta)	n/a	-6.8	L
PDGFRA (PDGFR alpha)	Km app	-6.8	Z
NEK9	Km app	-7.2	Z
PDK1	Km app	-7.3	Z
RPS6KB1 (p70S6K)	Km app	-7.8	Z
STK38L (NDR2)	n/a	-8.4	L
ABL1 G250E	Km app	-8.5	Z
PRKD1 (PKC mu)	Km app	-8.5	Z
CDK5/p35	Km app	-8.6	Z
PDGFRA T674I	Km app	-8.9	Z
BLK	Km app	-8.9	Z
RET V804L	Km app	-9.1	Z
PRKD2 (PKD2)	Km app	-9.1	Z
CSNK1G2 (CK1 gamma 2)	Km app	-9.3	Z
ROCK1	Km app	-9.5	Z
PRKCB2 (PKC beta II)	Km app	-9.6	Z
CSNK1G1 (CK1 gamma 1)	Km app	-9.9	Z
ABL2 (Arg)	Km app	-10.3	Z
STK3 (MST2)	Km app	-10.3	Z
CAMK4 (CaMKIV)	Km app	-10.4	Z
MKNK1 (MNK1)	Km app	-10.7	Z
CSNK1E (CK1 epsilon)	Km app	-10.9	Z
STK25 (YSK1)	Km app	-11.4	Z
FLT3 ITD	n/a	-11.6	L
NEK4	Km app	-11.6	Z
CHEK2 (CHK2)	Km app	-11.7	Z
MELK	Km app	-11.8	Z
LTK (TYK1)	Km app	-13.1	Z
MST1R (RON)	Km app	-13.4	Z
JAK2 JH1 JH2 V617F	Km app	-13.8	Z
EPHA8	Km app	-13.8	Z
EGFR (ErbB1) T790M L858R	Km app	-14.1	Z
MET M1250T	Km app	-14.5	Z
EGFR (ErbB1) L858R	Km app	-15.0	Z
GSG2 (Haspin)	Km app	-15.0	A
MET (cMet)	Km app	-15.1	Z
PLK3	Km app	-18.2	Z
NEK6	Km app	-19.8	Z
IRAK4	Km app	-20.5	Z
KIT T670I	Km app	-24.4	Z
PAK3	Km app	-42.6	Z

Supplemental Table 1. NU5455 kinase selectivity. The activity of NU5455 (1 μM) against 345 wild-type and 56 mutant kinases was profiled using the SelectScreen Broad Assay Panel (Life Technologies). A = Adapta activity assay, L = LanthaScreen binding assay, Z = Z'LYTE activity assay. The ATP concentration used was the Km_{app}, 10 or 100 μM, or was not applicable (n/a) for the LanthaScreen binding assay. The percentage values given refer to inhibition for the Adapta and Z-LYTE activity assays, and displacement for the LanthaScreen binding assay, and are the mean of two points.

PI3K Class	Kinase	NU5455 IC_{50} (nM)	Fold selectivity for DNA-PK
**Class IV (PIKK)**	DNA-PK	8.2 ± 0.8	-
	ATM	>10 000	>1220
	ATR	>10 000	>1220
	mTOR	4058 ± 1117	495
**Class III**	Vps34	71.0 ± 19	8.7
**Class II**	PI3K-C2α	>10 000	>1220
	*PI3K-C2β	*1956 ± 424	*239
	PI3K-C2γ	>10 000	>1220
**Class IB**	p110γ	>10 000	>1220
**Class IA**	p110α	1870 ± 216	228
	p110β	9320 ± 1245	1137
	p110δ	276 ± 22	33.7

Supplemental Table 2. NU5455 potency and selectivity across the PI3K kinase family. The *in vitro* potency and fold-selectivity of 1 µM NU5455 for DNA-PK versus other PIKK and PI3K family members was assessed by SelectScreen Profiling (Life Technologies). The ATP concentration was the Kmapp except for * where the ATP concentration was 10 µM (mean ± SEM, n=4-7).

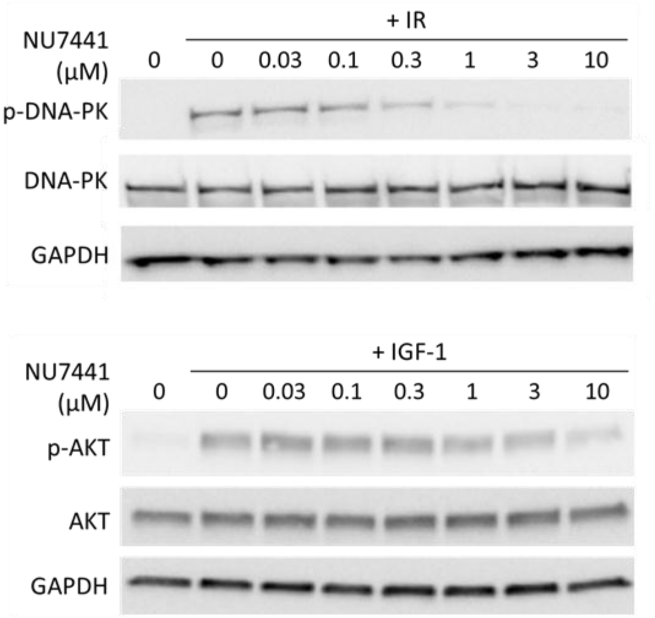

Supplemental Figure 2. NU7441 inhibits DNA-PK and AKT phosphorylation in MCF7 cells. Western blots demonstrating changes in phospho-DNA-PK Ser^{2056} 30 minutes post-treatment with 10 Gy IR, and changes in phospho-AKT Ser^{473} as a marker of PI-3K activity 30 minutes post-treatment with 50 ng/ml IGF-1, in MCF7 cells pre-treated with vehicle or NU7441 for 1 hour. Blots are representative of three independent experiments.

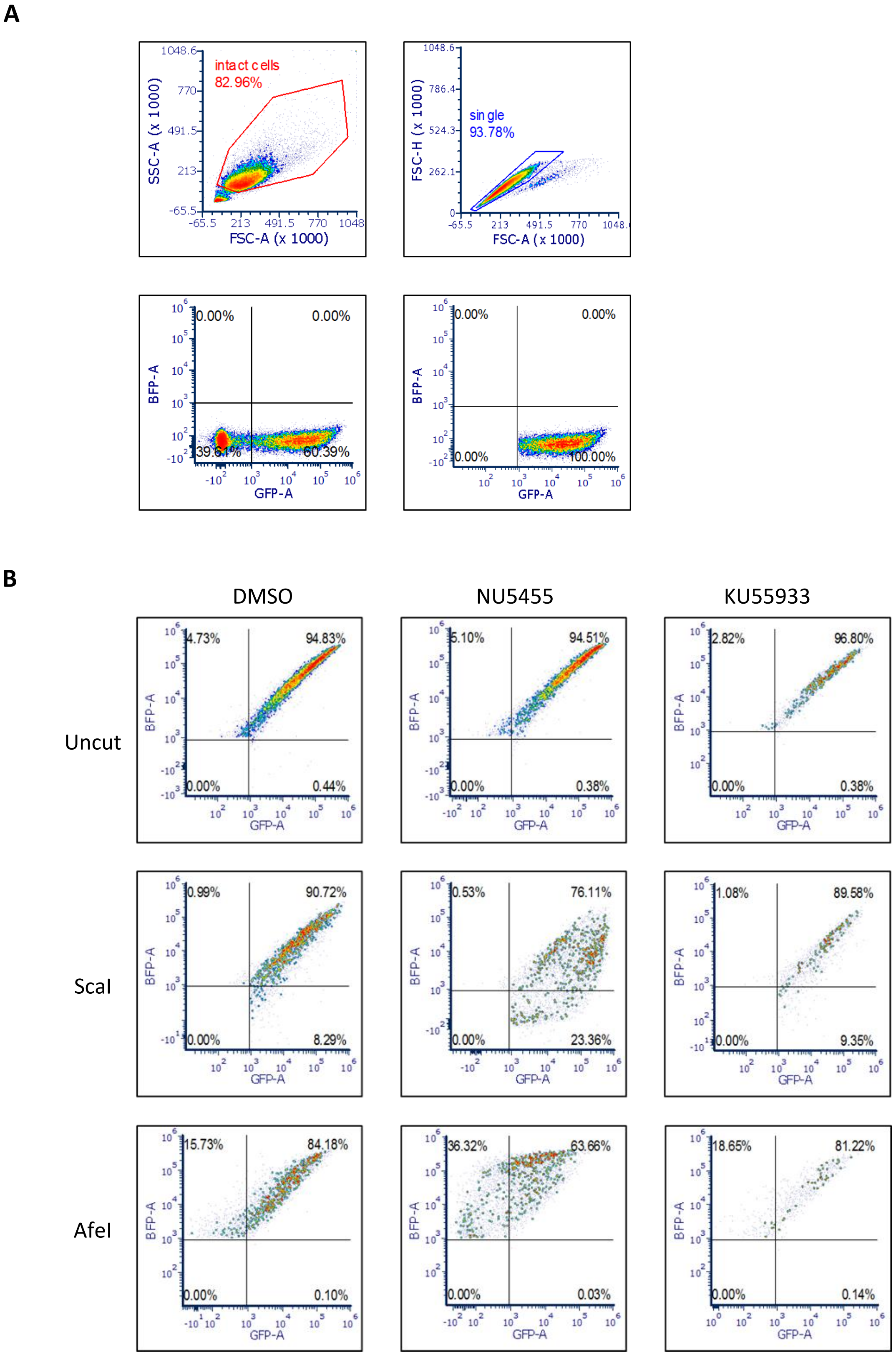

Supplemental Figure 3. NU5455 but not the ATM inhibitor KU55933 inhibits NHEJ-mediated DSB repair in HEK293T cells. A plasmid repair assay enabled quantification of NHEJ-mediated DSB repair in HEK293T cells by measurement of the relative proportions of BFP and GFP by flow cytometry. Cells were transfected with intact or linearized (AfeI or ScaI restriction endonuclease treated) plasmid DNA and treated with DMSO, NU5455 (1 µM) or the ATM inhibitor KU55933 (10 µM) for 24 hours. (**A**) Firstly, intact and single cell populations were defined by gating on the side-scatter and forward-scatter. To define the transfected cell population (cells expressing one or two fluorescent proteins), quadrant gating was used with a threshold cut off of $1x10^3$ fluorescent intensity units for both GFP and BFP signals. (**B**) FACS plots of HEK293T cells transfected with the intact (uncut) or linearized (AfeI or ScaI restriction endonuclease treated) plasmid DNA and treated with DMSO, NU5455 or KU55933 for 24 hours. Data are representative of three independent experiments.

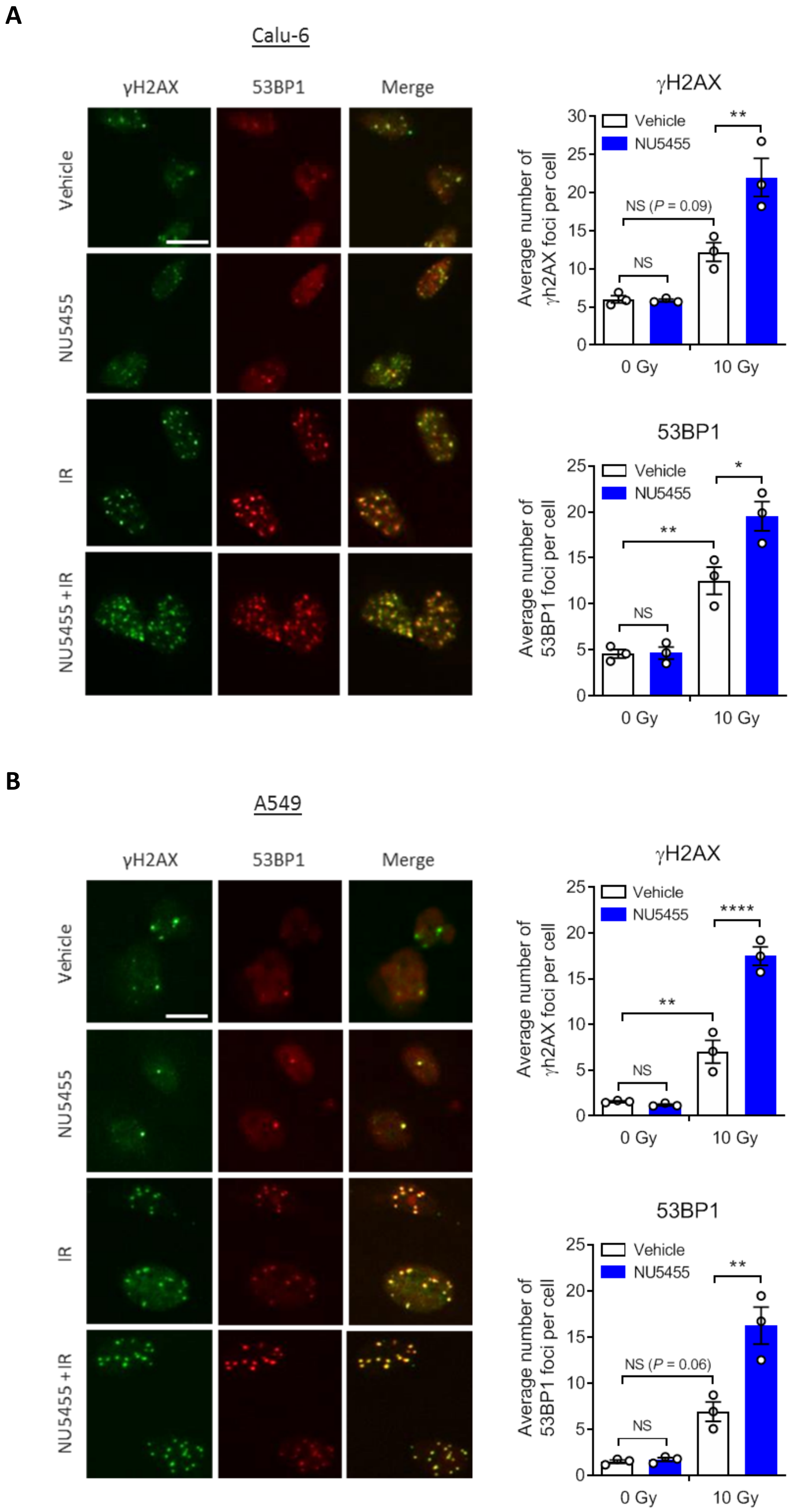

Supplemental Figure 4. NU5455 augments radiation-induced 53BP1 and γH2AX foci numbers in Calu-6 and A549 cells. Representative images of γH2AX and 53BP1 foci in (**A**) Calu-6 and (**B**) A549 cells treated with NU5455 (5 µM) 1 hour prior to irradiation (10 Gy) and fixed 5 hours post-irradiation. Foci quantitation was performed on 400 nuclei per sample. Scale bars = 10 µm. Data represent the mean ± SEM from three independent experiments, and statistical significance was assessed using a 1-way ANOVA. NS = not significant, * $P < 0.05$, ** $P < 0.01$, **** $P < 0.0001$.

A

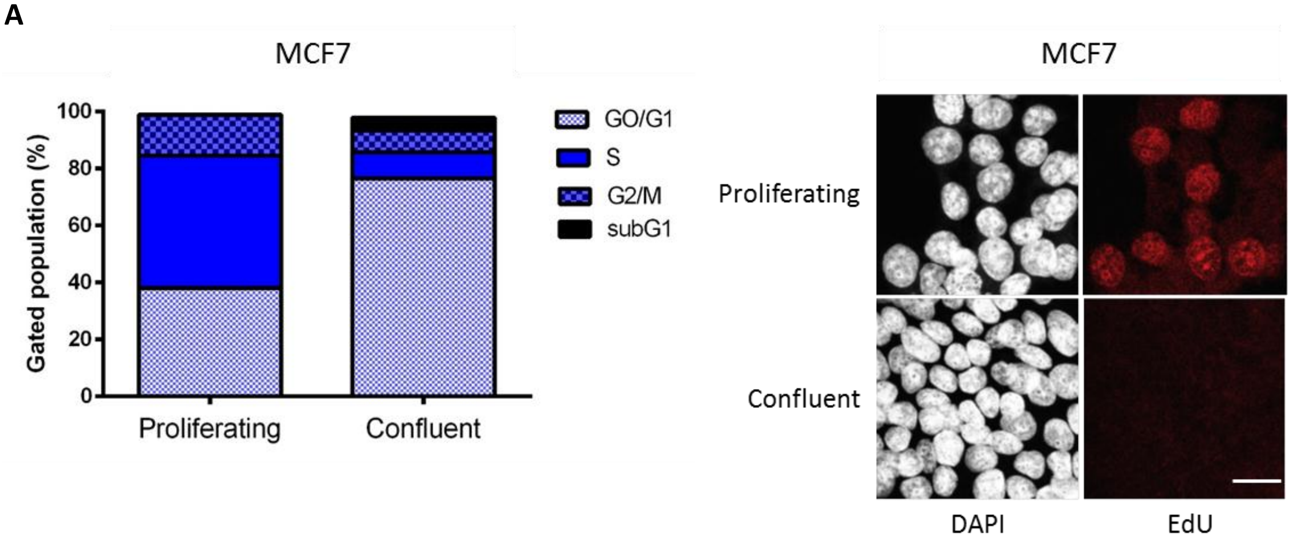

B

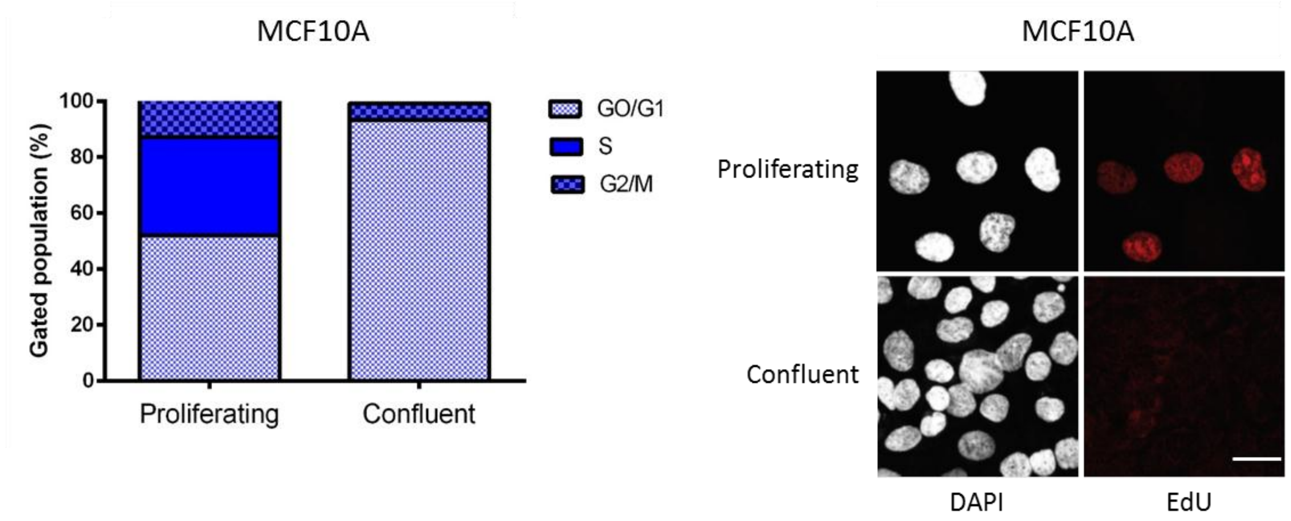

Supplemental Figure 5. Cell cycle profile analysis and fluorescence microscopy to distinguish proliferating and confluent cells. Representative flow cytometry data and images for MCF7 (**A**) and MCF10A (**B**) cells after pulse-labelling with 10 µM EdU to detect proliferating cells. Cells were analysed either when proliferating or after being allowed to reach confluency (as detailed in the Supplemental Methods). Data and images shown are representative of four independent experiments. Scale bars = 20 µm.

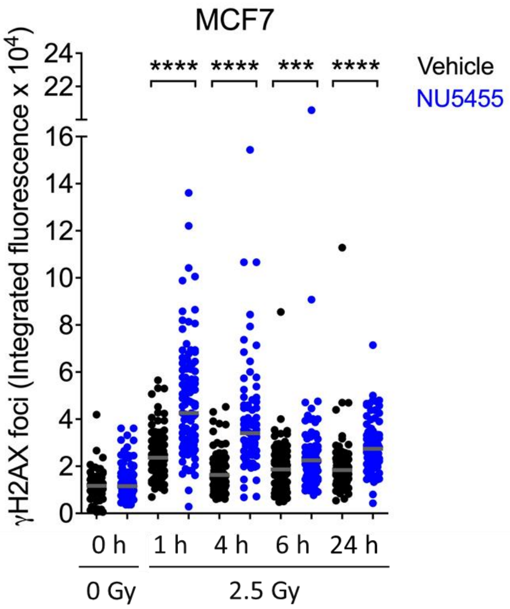

Supplemental Figure 6. NU5455 augments radiation-induced γH2AX foci integrated fluorescence in MCF7 cells. Separate replicate experiment to determine the integrated total nuclear fluorescence of γH2AX foci in MCF7 cells pre-treated with NU5455 (1 µM) for 1 hour and fixed 0 to 24 hours post-irradiation (2.5 Gy) (see also Fig. 2E). ≥ 50 cells analysed per treatment group. Significant enhancement by NU5455 is denoted by *** $P < 0.001$, **** $P < 0.0001$ (Mann Whitney U test).

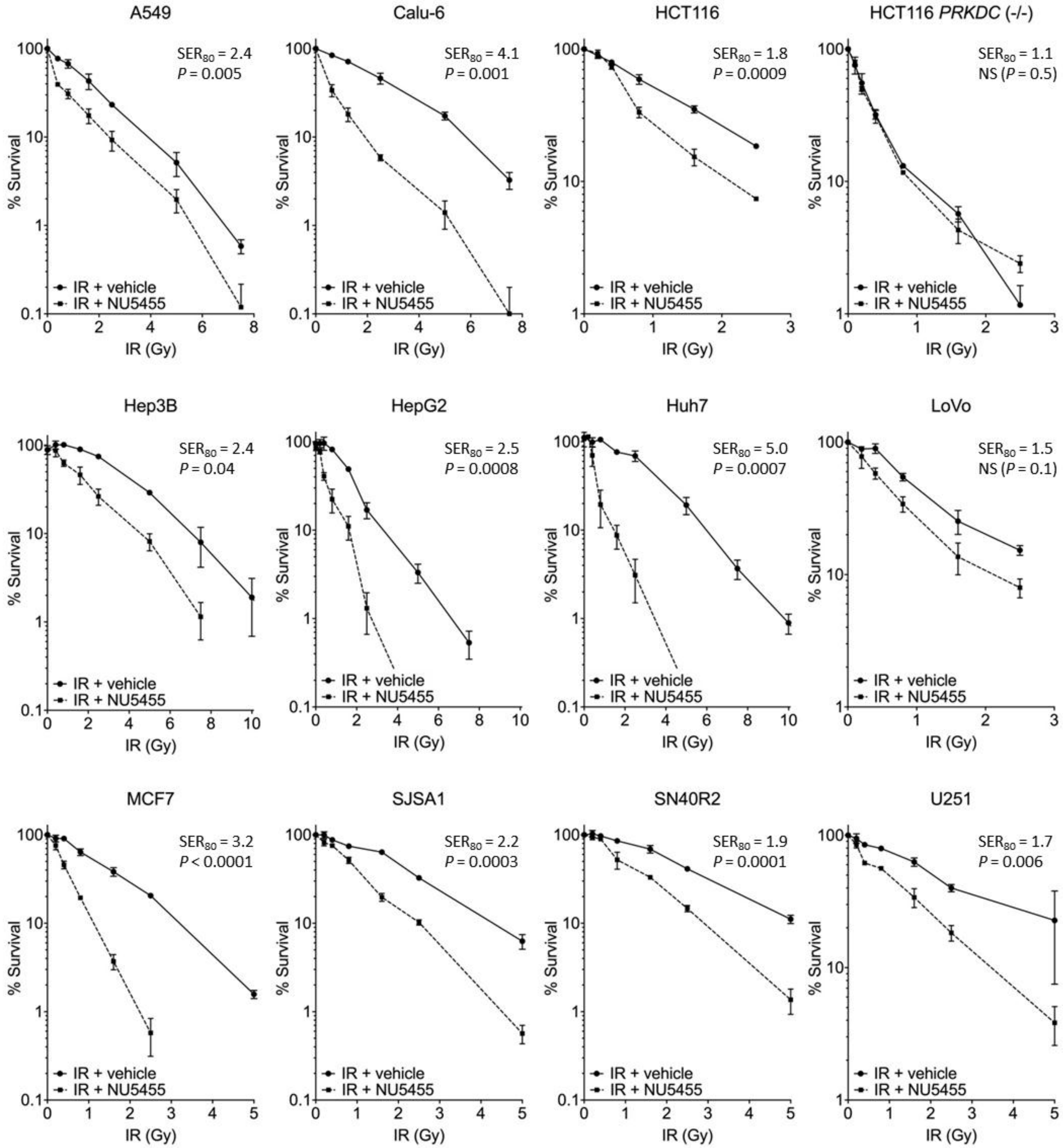

Supplemental Figure 7. NU5455 sensitizes human tumor cell lines to ionizing radiation. Clonogenic survival assays of human tumor cell lines pre-treated with vehicle or NU5455 (1 µM) for 1 hour prior to irradiation. Cells were then incubated with NU5455 or vehicle for 24 hours post-irradiation prior to reseeding into drug-free media. Graphs represent the mean ± SEM from three independent experiments. SER_{80} = the sensitizing enhancement ratio 80, which is the ratio between radiation doses with and without NU5455 that induced an 80% inhibition of clonogenic cell survival. Statistical significance was assessed using an unpaired *t*-test, with values being shown to one significant figure.

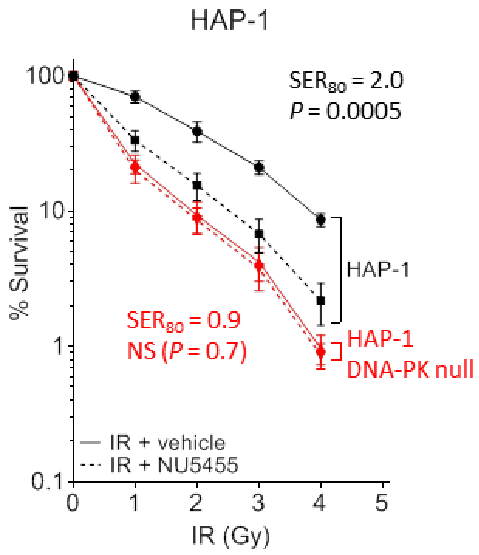

Supplemental Figure 8. NU5455 sensitizes HAP-1 cells but not HAP-1 DNA-PK null cells to ionizing radiation. Clonogenic survival of HAP-1 DNA-PK proficient or deficient cells treated with NU5455 (5 µM) for 1 hour prior to irradiation, and for a further 24 hours prior to continued incubation in drug-free medium. Graph represents the mean ± SEM from three independent experiments. SER_{80} = the sensitizing enhancement ratio 80, which is the ratio between radiation doses with and without NU5455 that induced an 80% inhibition of clonogenic cell survival. Statistical significance was assessed using an unpaired *t*-test, with values being shown to one significant figure.

Cell Line	Origin	*TP53* status	Details	Nutlin-3a GI_{50} (µM)	Reference source
A549	Human	WT		2.4, 2.9	COSMIC
B16F10	Mouse	WT		5.7 ± 1.8	(1)
CT26	Mouse	WT			(2)
HCT116	Human	WT		2.1 ± 0.71	COSMIC
HEK293T	Human	WT			(3)
HepG2	Human	WT		1.0 ± 0.18	COSMIC
LoVo	Human	WT			COSMIC
M2-10B4	Mouse	WT		3.3 ± 0.64	
MCF7	Human	WT		2.3 ± 0.20	COSMIC
MCF10a	Human	WT		5.1 ± 2.8	(4)
NHDF	Human	WT		2.7	(5)
NIH3T3	Mouse	WT		4.0 ± 1.1	(6)
SJSA-1	Human	WT		1.9 ± 0.19	COSMIC
3T3-Swiss	Mouse	WT			(7)
Calu6	Human	MT	c.586C>T Nonsense	> 10, > 30	COSMIC
HCT116 *TP53* $^{-/-}$	Human	Del		> 10, 29, 27	(8)
Hep3B	Human	Del		> 10, >10, > 30	(9)
Huh7	Human	MT	c.659A>G Missense	> 10, 27	COSMIC
SN40R2	Human	MT	c.285G>A Missense	> 30	(10)
4T1	Mouse	Null		>30, >30	(11)
U251	Human	MT	c.818G>A Missense		COSMIC

Supplemental Table 3. *TP53* status of cell lines used. Whether cells had been previously characterized for the presence of functional wild-type p53 (WT), or loss of p53 function through a known *TP53* gene mutation (MT), deletion (Del), or an absence of protein (Null), was determined through interrogation of COSMIC (https://cancer.sanger.ac.uk/cosmic) or the literature. In addition, the growth inhibitory activity of Nutlin-3a, an MDM2-p53 inhibitor which reactivates p53 in wild-type cells, but not in those with compromised p53 function, was determined in a number of cell lines by measurement of the GI_{50} value following a 72 hour incubation with the compound (see Supplemental Methods). GI_{50} values shown represent either individual determinations or the mean with standard deviation for experiments with ≥3 defined values. References: (1) Melnikova VO et al., *Oncogene*, 2004;23:2347-2356. (2) Castle JC et al., *BMC Genomics*, 2014;15:190. (3) Sun L et al., *Acta Biochim Biophys Sin (Shanghai)*, 2010;42:230-235. (4) Weiss MB et al., *Oncogene*, 2010;29:4715-4724. (5) Cam M et al., *J Biol Chem*, 2014:289:4083-4094. (6) Tovar C et al., *Proc Natl Acad Sci USA*, 2006;103:1888-1893. (7) Ho CC et al., *Oncotarget*, 2010;1:583-595. (8) horizondiscovery.com (9) Bressac B et al., *Proc Natl Acad Sci USA*, 1990;87:1973-1977. (10) Drummond CJ et al., *Oncotarget*, 2016;7:46203-46218. (11) Yerlikaya A, Erin N. *Int J Mol Med*, 2008;22:817-823.

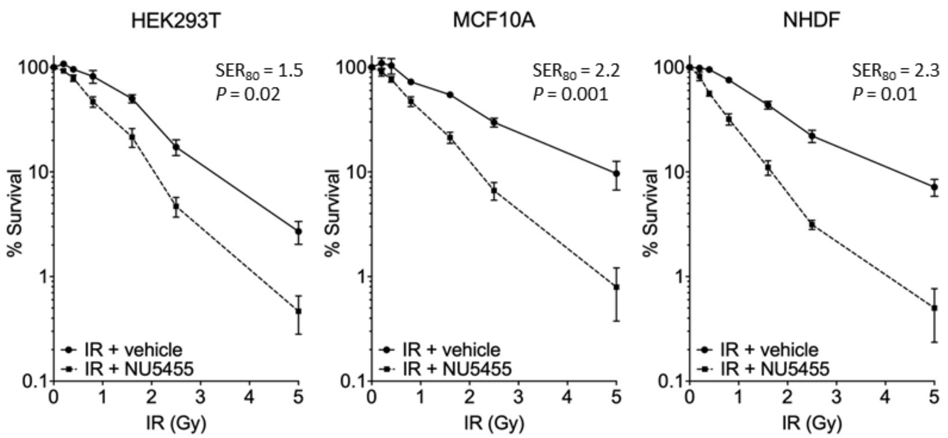

Supplemental Figure 9. NU5455 sensitizes human non-tumor cell lines to ionizing radiation. Clonogenic survival assays of human non-tumor cell lines pre-treated with vehicle or NU5455 (1 µM) for 1 hour prior to irradiation. Cells were then incubated with NU5455 or vehicle for 24 hours post-irradiation prior to reseeding into drug-free media. Graphs represent the mean ± SEM from three independent experiments. SER_{80} = the sensitizing enhancement ratio 80, which is the ratio between radiation doses with and without NU5455 that induced an 80% inhibition of clonogenic cell survival. Statistical significance was assessed using an unpaired *t*-test, with values being shown to one significant figure.

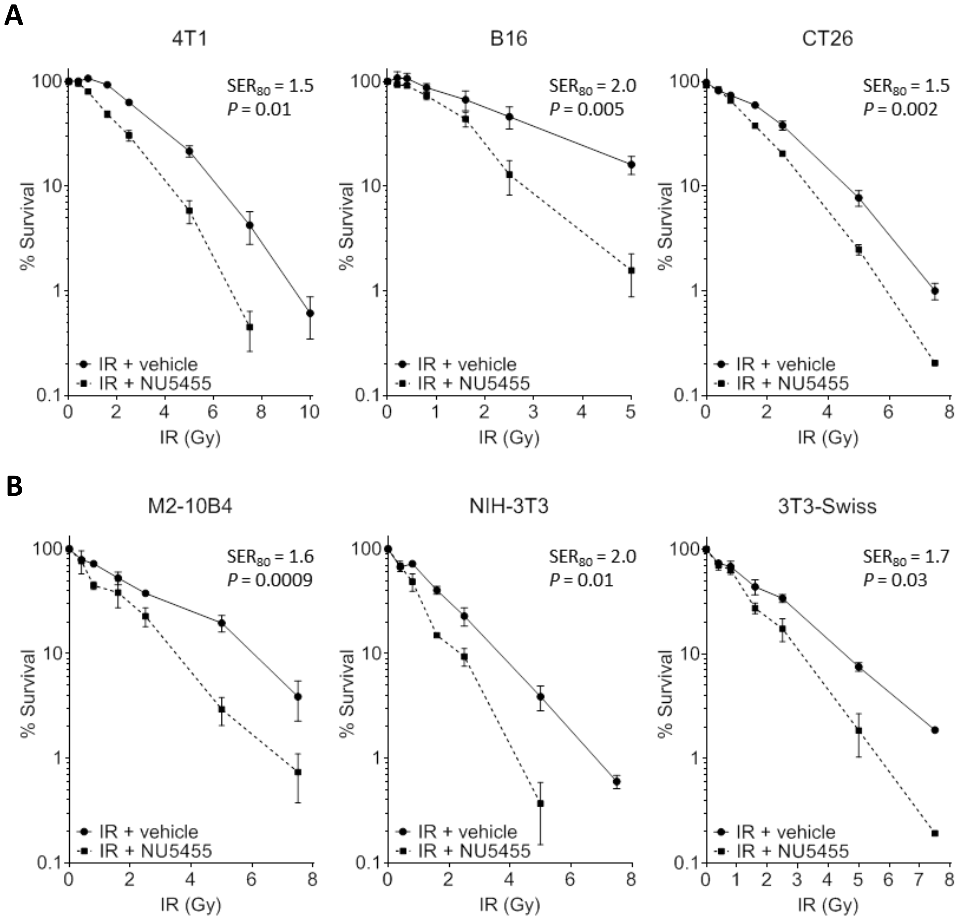

Supplemental Figure 10. NU5455 sensitizes murine tumor and non-tumor cell lines to ionizing radiation. Clonogenic survival assays of murine tumor (**A**) and non-tumor (**B**) cell lines pre-treated with vehicle or NU5455 (1 µM) for 1 hour prior to irradiation. Cells were then incubated with NU5455 or vehicle for 24 hours post-irradiation prior to reseeding into drug-free media. Graphs represent the mean ± SEM from three independent experiments. SER_{80} = the sensitizing enhancement ratio 80, which is the ratio between radiation doses with and without NU5455 that induced an 80% inhibition of clonogenic cell survival. Statistical significance was assessed using an unpaired *t*-test, with values being shown to one significant figure.

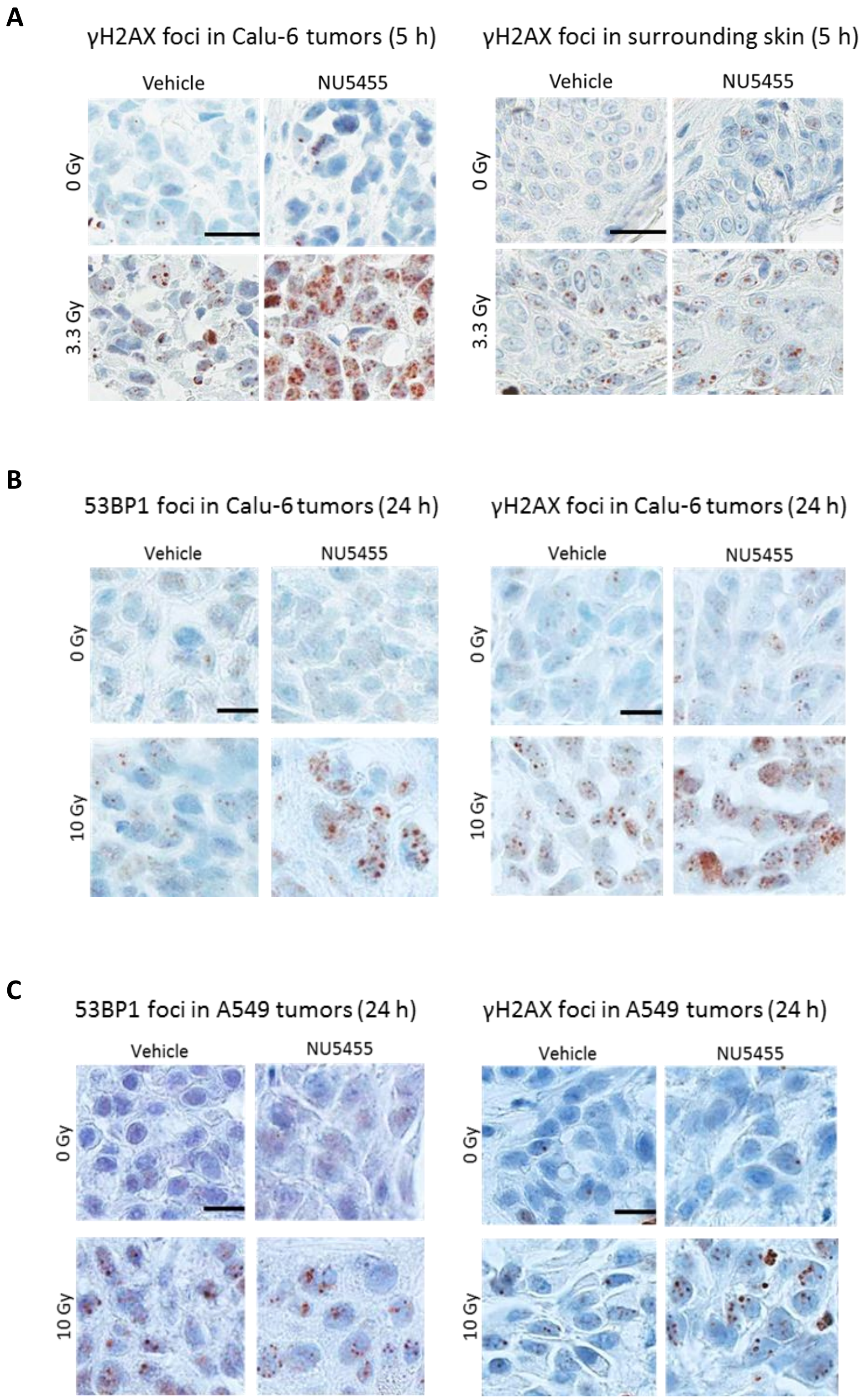

Supplemental Figure 11. NU5455 increases the number of radiation-induced 53BP1 and γH2AX foci in Calu-6 and A549 subcutaneous xenografts, but does not significantly augment γH2AX foci number in the surrounding skin. (**A**) Representative IHC images of γH2AX foci in subcutaneous Calu-6 tumors and the surrounding skin from mice treated with NU5455 (30 mg/kg, *p.o.*) or vehicle 30 minutes prior to localized radiation (0 Gy or 3.3 Gy). Samples were collected 5 hours post-irradiation. Scale bars = 25 µm. (**B**) Representative images of γH2AX and 53BP1 IHC staining in Calu-6 subcutaneous xenografts from mice treated with NU5455 (30 mg/kg, *p.o.*) or vehicle 30 minutes prior to localized radiation (0 Gy or 10 Gy). Samples were collected 24 hours post-irradiation. Scale bars = 12.5 µm. (**C**) Representative images of 53BP1 and γH2AX IHC staining in A549 subcutaneous xenografts from mice treated with NU5455 (30 mg/kg, *p.o.*) or vehicle 30 minutes prior to localized radiation (0 Gy or 10 Gy). Samples were collected 24 hours post-irradiation. Scale bars = 12.5 µm.

A

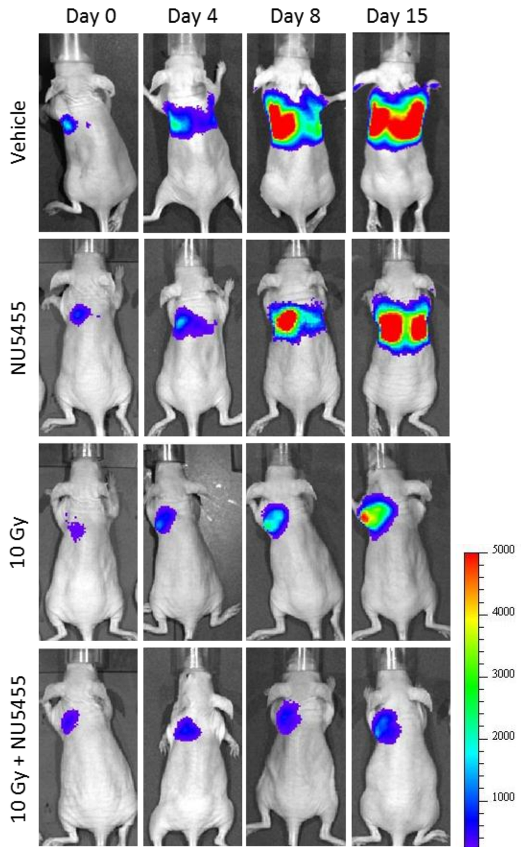

B

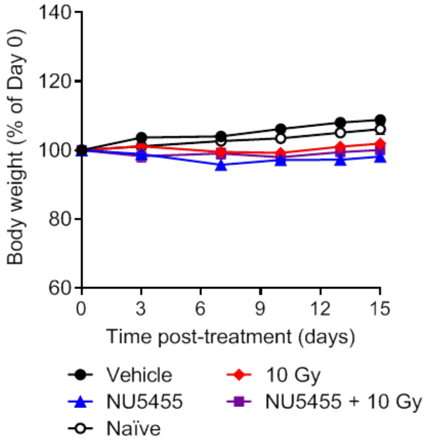

Supplemental Figure 12. NU5455 augments the effect of radiotherapy in orthotopically implanted Calu-6 lung tumors without influencing body weight. (**A**) Representative bioluminescent images of mice orthotopically implanted with luciferase-expressing Calu-6 cells and treated with NU5455 (30 mg/kg, *p.o.*) or vehicle 30 minutes prior to thoracic radiation (0 Gy or 10 Gy, 3-4 mice per treatment group) one week following implantation. Bioluminescent images were acquired using an IVIS Spectrum 0, 4, 8 and 15 days after treatment. Each set of images at Day 0 and Day 15 are reproduced from Figure 4B. (**B**) Percentage change in body weight over time. Data represent the mean ± SEM (3-4 mice per treatment group).

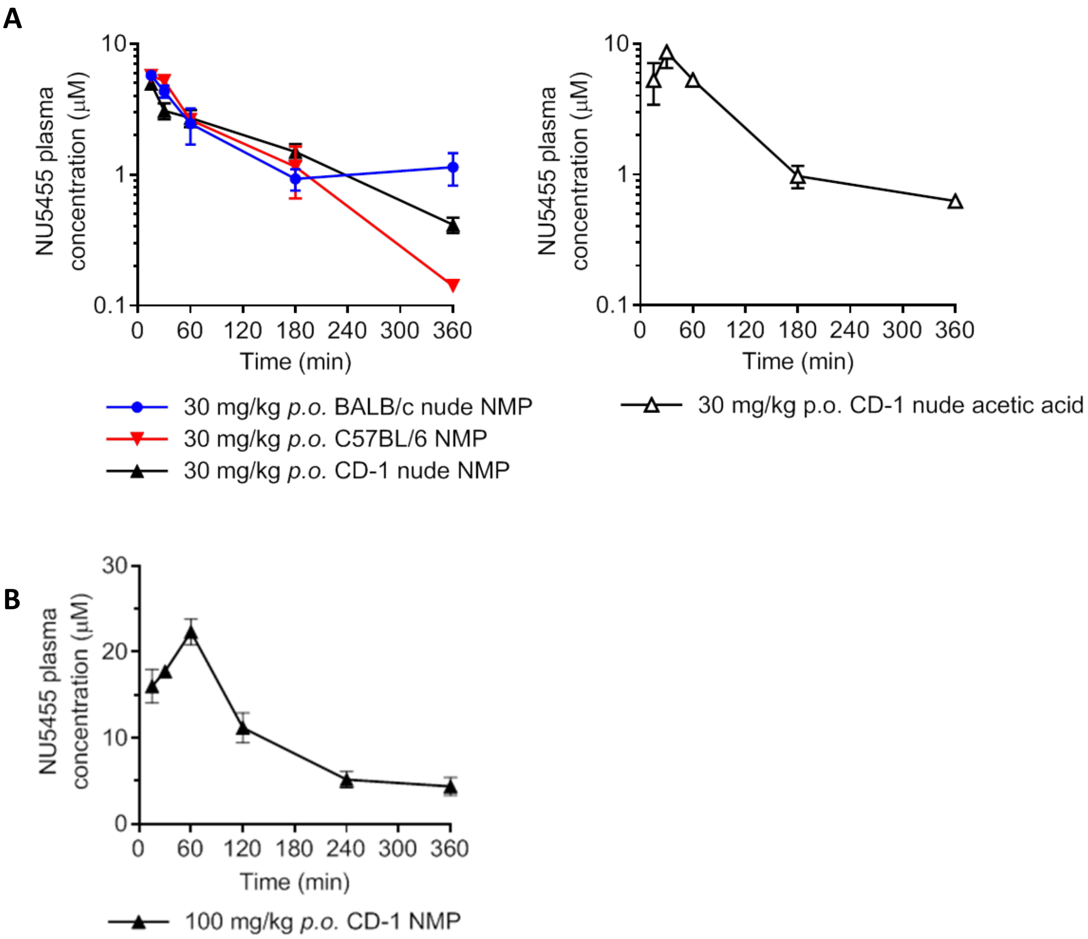

Supplemental Figure 13. Pharmacokinetic profiles of NU5455. (**A**) NU5455 plasma concentrations in BALB/c nude, C57BL/6 and CD-1 nude mice 15-360 minutes post-administration of 30 mg/kg NU5455 (*p.o.*) prepared in NMP:Encapsin (30% aq):PEG4000 (1:6:3 v/v/v) (NMP), or 1% (v/v) acetic acid. (**B**) NU5455 plasma concentrations in CD-1 mice 15-360 minutes post-administration of 100 mg/kg NU5455 (*p.o.*) prepared in NMP:Encapsin (30% aq):PEG4000 (1:6:3 v/v/v) (NMP). All graphs represent mean ± SEM (3 mice per treatment group).

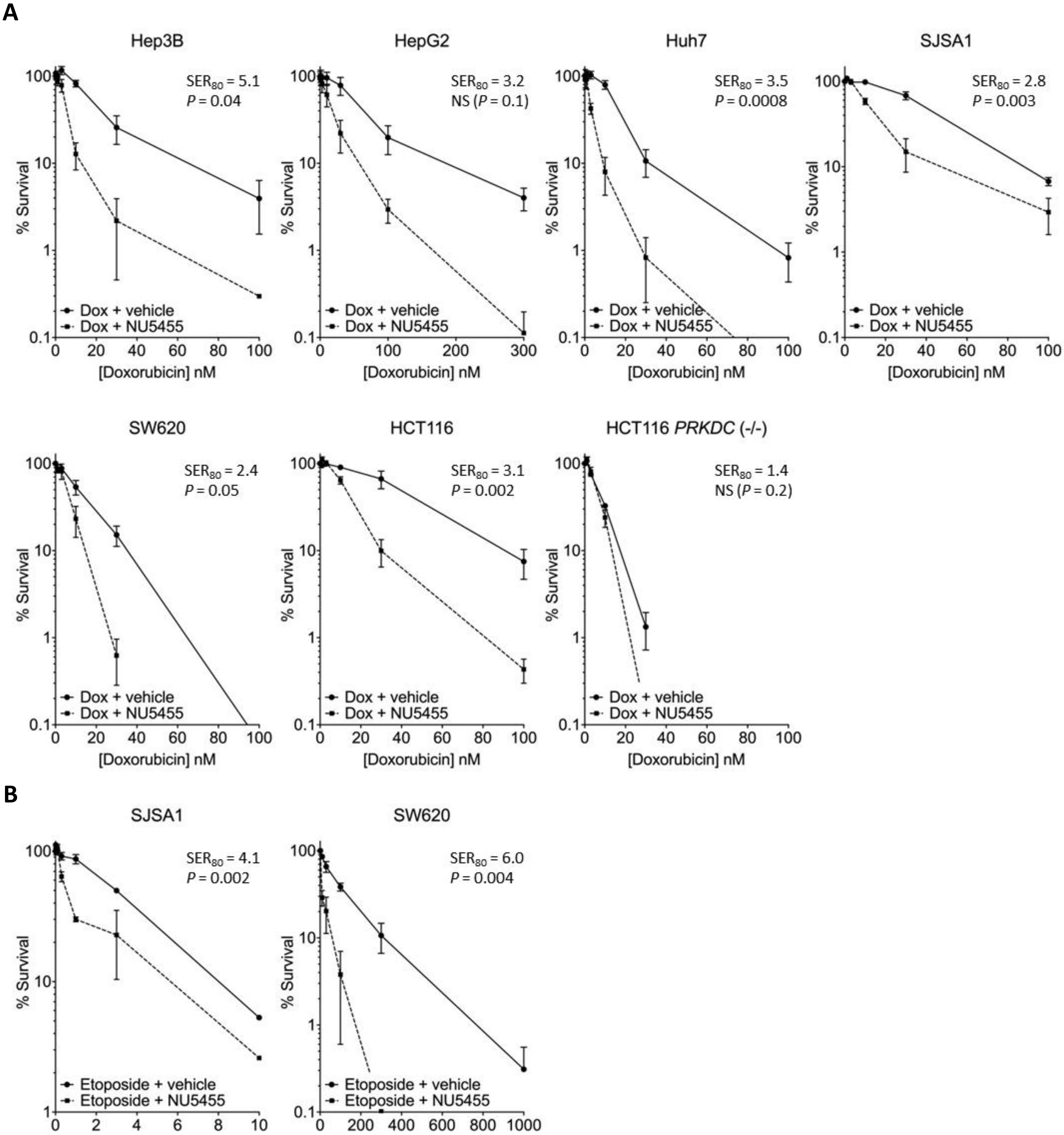

Supplemental Figure 14. NU5455 sensitizes human tumor cell lines to topoisomerase II inhibitors. Clonogenic survival assays of human tumor cell lines pre-treated with vehicle or NU5455 (1 μM) for 1 hour prior to the addition of doxorubicin (**A**) or etoposide (**B**). Cells were incubated in the presence of both treatments for 24 hours before replating into drug-free media. Graphs represent the mean ± SEM from three independent experiments. SER_{80} = the sensitizing enhancement ratio 80, which is the ratio between radiation doses with and without NU5455 that induced an 80% inhibition of clonogenic cell survival. Statistical significance was assessed using an unpaired *t*-test, with values being shown to one significant figure. Data shown for Huh7 with doxorubicin (**A**) and SJSA1 with etoposide (**B**) are reproduced from Figure 6A.

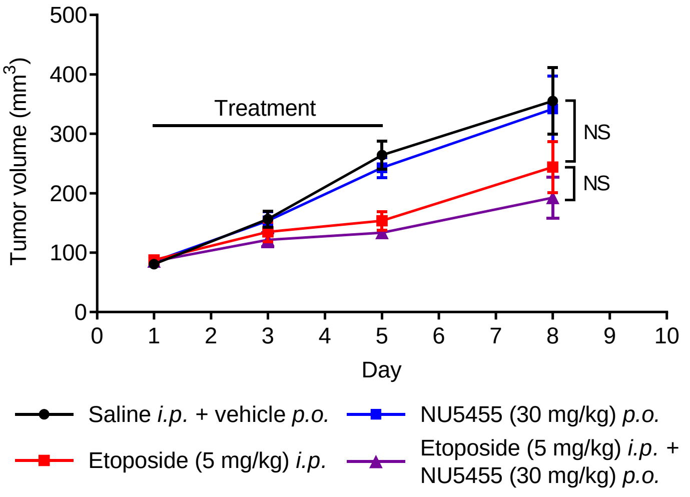

Supplemental Figure 15. NU5455 (30 mg/kg) does not significantly enhance the anti-tumor activity of etoposide (5 mg/kg) in SJSA1 subcutaneous xenografts. Tumor volumes from mice bearing SJSA1 subcutaneous xenografts treated with NU5455 (30 mg/kg, *p.o.*) ± etoposide (5 mg/kg, *i.p.*) daily for 5 days. Data represent mean ± SEM (10 mice per treatment group). NS = not significant (unpaired *t*-test).

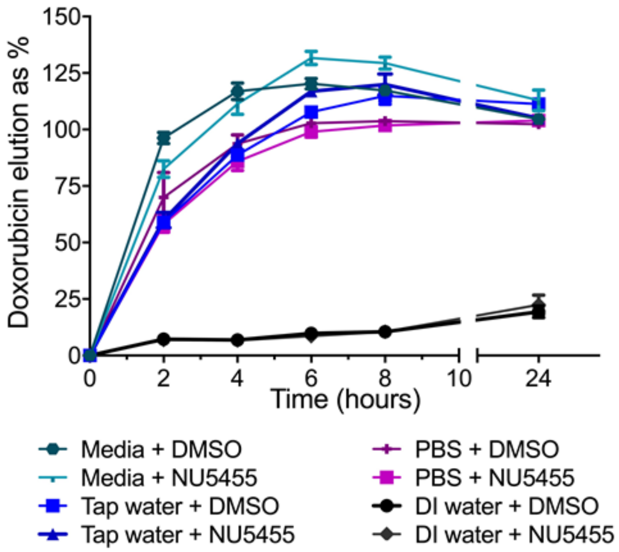

Supplemental Figure 16. NU5455 does not influence the rate of doxorubicin elution from DC *M1* beads. The *in vitro* elution of doxorubicin from DC *M1* beads loaded at 25 mg/ml into cell culture medium, water (tap or deionized) or PBS containing 10 µM NU5455 or DMSO. Elution was determined at 37°C using a doxorubicin autofluorescence assay and quantified against a free doxorubicin standard curve. The graph depicts mean doxorubicin elution as a percentage of mean autofluorescence at 48 hours ± SEM. Data are representative of three independent experiments.

A

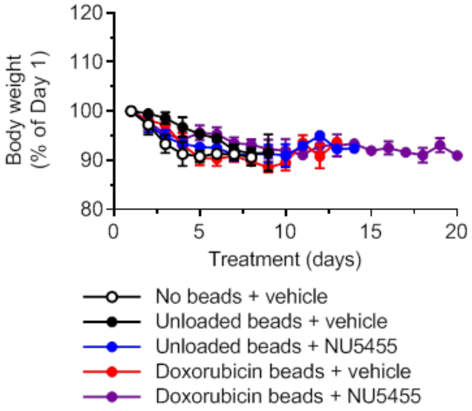

B

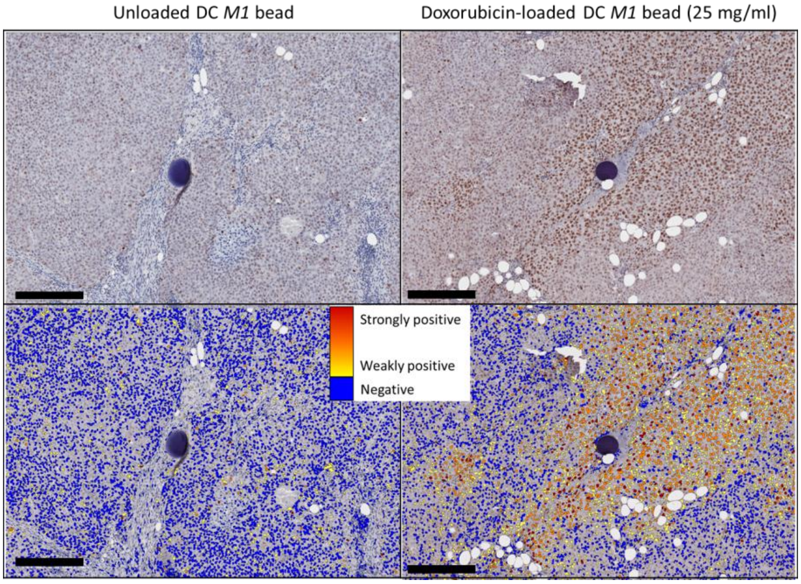

C

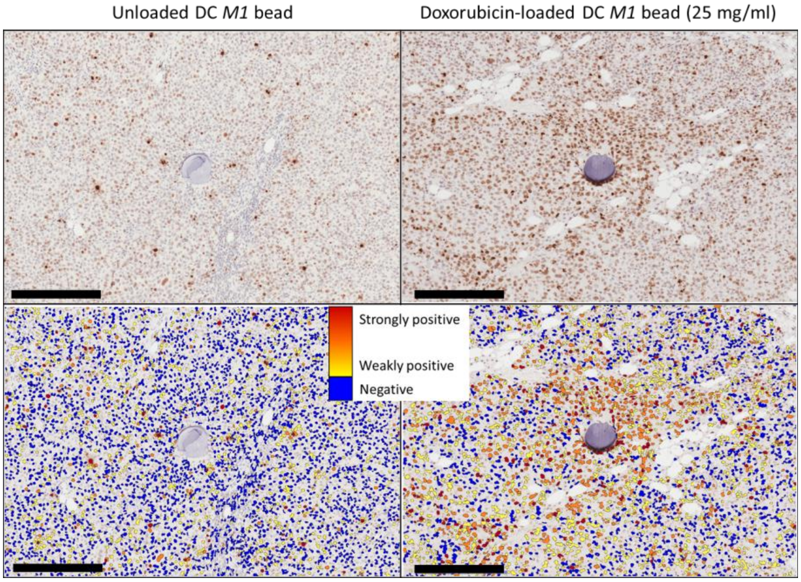

Supplemental Figure 17. NU5455 augments localized doxorubicin chemotherapy in HCC tumor xenografts without increased toxicity. (**A**) Percentage change in body weight over time of mice treated with 5 µl unloaded or doxorubicin-loaded DC *M1* beads (25 mg/ml) implanted *via* an intra-tumoral injection, followed by twice-daily dosing with NU5455 (30 mg/kg, *p.o.*) or vehicle control (9 hour dosing interval, 6 mice per group). Representative images of (**B**) pDNA-PKcs Ser^{2056} or (**C**) γH2AX staining in Huh7 xenografts treated with unloaded or doxorubicin-loaded DC *M1* beads (25 mg/ml), with the nuclear positivity algorithm overlaid. Key inset. Scale bars = 300 µm. Images in (**B**) and (**C**) presented without the nuclear positivity algorithm overlaid represent larger areas of the images shown originally in Figure 7F.

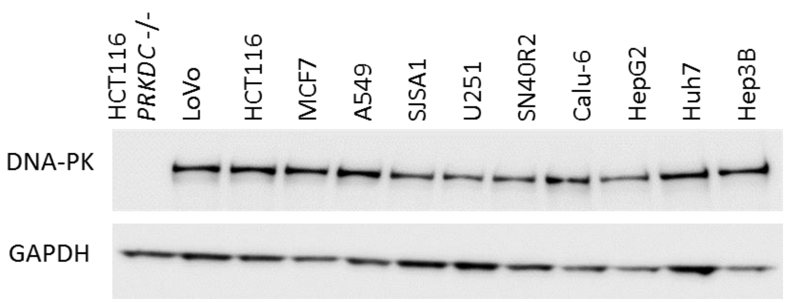

Supplemental Figure 18. Basal DNA-PK protein expression across a panel of human tumor cell lines. Western blot depicting total DNA-PK protein expression in 12 human tumor cell lines, in order of intrinsic radiosensitivity in clonogenic survival assays as presented in Figure 2F. Blot is representative of two independent experiments.

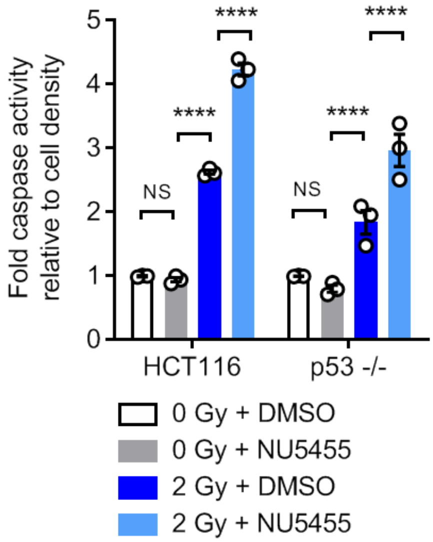

Supplemental Figure 19. NU5455 increases radiation-induced apoptosis in both HCT116 parental and *TP53* null cell lines. Fold caspase activity in HCT116 parental and p53 null (-/-) cells 72 hours following pre-treatment with NU5455 (1 μM) or DMSO for 1 hour prior to irradiation (0 or 2 Gy). Fold caspase activity was normalized to cell density determined by SRB staining. Data represent the mean ± SEM from three independent experiments. NS = not significant, **** $P < 0.0001$ (2-way ANOVA).
